# The evolutionary convergence of mid-Mesozoic lacewings and Cenozoic butterflies

**DOI:** 10.1098/rspb.2015.2893

**Published:** 2016-02-10

**Authors:** Conrad C. Labandeira, Qiang Yang, Jorge A. Santiago-Blay, Carol L. Hotton, Antónia Monteiro, Yong-Jie Wang, Yulia Goreva, ChungKun Shih, Sandra Siljeström, Tim R. Rose, David L. Dilcher, Dong Ren

**Affiliations:** 1 College of Life Sciences, Capital Normal University, Beijing 100048, People's Republic of China; 2 Department of Paleobiology, National Museum of Natural History, Smithsonian Institution, Washington, DC 20013, USA; 3 Department of Mineral Sciences, National Museum of Natural History, Smithsonian Institution, Washington, DC 20013, USA; 4 Department of Entomology and BEES Program, University of Maryland, College Park, MD 20742, USA; 5 State Key Laboratory of Biocontrol, Key Laboratory of Biodiversity Dynamics and Conservation of Guangdong Higher Education Institute, College of Ecology and Evolution, School of Life Sciences, Sun Yat-sen University, Guangzhou 510275, People's Republic of China; 6 Geoscience Museum, Shijiazhuang University of Economics, Shijiazhuang 050031, People's Republic of China; 7 Department of Crop and Agroenvironmental Sciences, University of Puerto Rico, Mayagüez, PR 00681, USA; 8 National Centre for Biotechnology Information, National Library of Medicine, Bethesda, MD 20892, USA; 9 Department of Ecology and Evolutionary Biology, Yale University, New Haven, CT 06511, USA; 10 Department of Biological Sciences, National University of Singapore, Singapore 117543, Singapore; 11 Yale-NUS College, Singapore 138614, Singapore; 12 Jet Propulsion Laboratory, National Aeronautics and Space Administration, Pasadena, CA 91125, USA; 13 Department of Chemistry, Materials and Surfaces, SP Technical Research Institute of Sweden, Borås 51115, Sweden; 14 Geophysical Laboratory, Carnegie Institution of Washington, Washington, DC 20015, USA; 15 Departments of Geology and Biology, Indiana University, Bloomington, IN 47405, USA

**Keywords:** angiosperms, gymnosperms, Kalligrammatidae, Papilionoidea, tubular proboscis, wing eyespots

## Abstract

Mid-Mesozoic kalligrammatid lacewings (Neuroptera) entered the fossil record 165 million years ago (Ma) and disappeared 45 Ma later. Extant papilionoid butterflies (Lepidoptera) probably originated 80–70 Ma, long after kalligrammatids became extinct. Although poor preservation of kalligrammatid fossils previously prevented their detailed morphological and ecological characterization, we examine new, well-preserved, kalligrammatid fossils from Middle Jurassic and Early Cretaceous sites in northeastern China to unravel a surprising array of similar morphological and ecological features in these two, unrelated clades. We used polarized light and epifluorescence photography, SEM imaging, energy dispersive spectrometry and time-of-flight secondary ion mass spectrometry to examine kalligrammatid fossils and their environment. We mapped the evolution of specific traits onto a kalligrammatid phylogeny and discovered that these extinct lacewings convergently evolved wing eyespots that possibly contained melanin, and wing scales, elongate tubular proboscides, similar feeding styles, and seed–plant associations, similar to butterflies. Long-proboscid kalligrammatid lacewings lived in ecosystems with gymnosperm–insect relationships and likely accessed bennettitalean pollination drops and pollen. This system later was replaced by mid-Cretaceous angiosperms and their insect pollinators.

## 1. Introduction

Lepidoptera and Neuroptera are members of two basal clades of Holometabola that separated *ca* 320 million years ago (Ma) during the mid-Carboniferous [1,2]. Although butterflies (Lepidoptera; Papilionoidea) are perhaps the most iconic group of insect pollinators [3], their earliest definitive fossils occur at the Palaeocene–Eocene boundary, 56 Ma [3]. Molecular studies of various family level ranks [4,5] suggest an earlier, Late Cretaceous origin at *ca* 80–70 Ma [5,6], considerably after the mid-Cretaceous (125–100 Ma) angiosperm radiation [7]. Butterflies are characterized by a distinctive ensemble of traits, such as diurnal behaviour, tubular (siphonate) mouthparts, wing eyespot patterns and wing scales [3,8,9]. These features appeared at the origin of the clade, allowing butterflies intimate association with more derived angiosperms during the Late Cretaceous and Palaeogene (80–23 Ma), and led to the coevolution and diversification of both groups [5,10]. Was this stereotypical assembly of butterfly features a one-time innovation uniquely associated with angiosperms? Or did the butterfly character-suite evolve in unrelated insect lineages with earlier gymnosperms? Here, we report on a distinctive clade of butterfly-like insects, Kalligrammatidae (Neuroptera), and explore their biological convergence with Papilionoidea.

**Figure 1.**
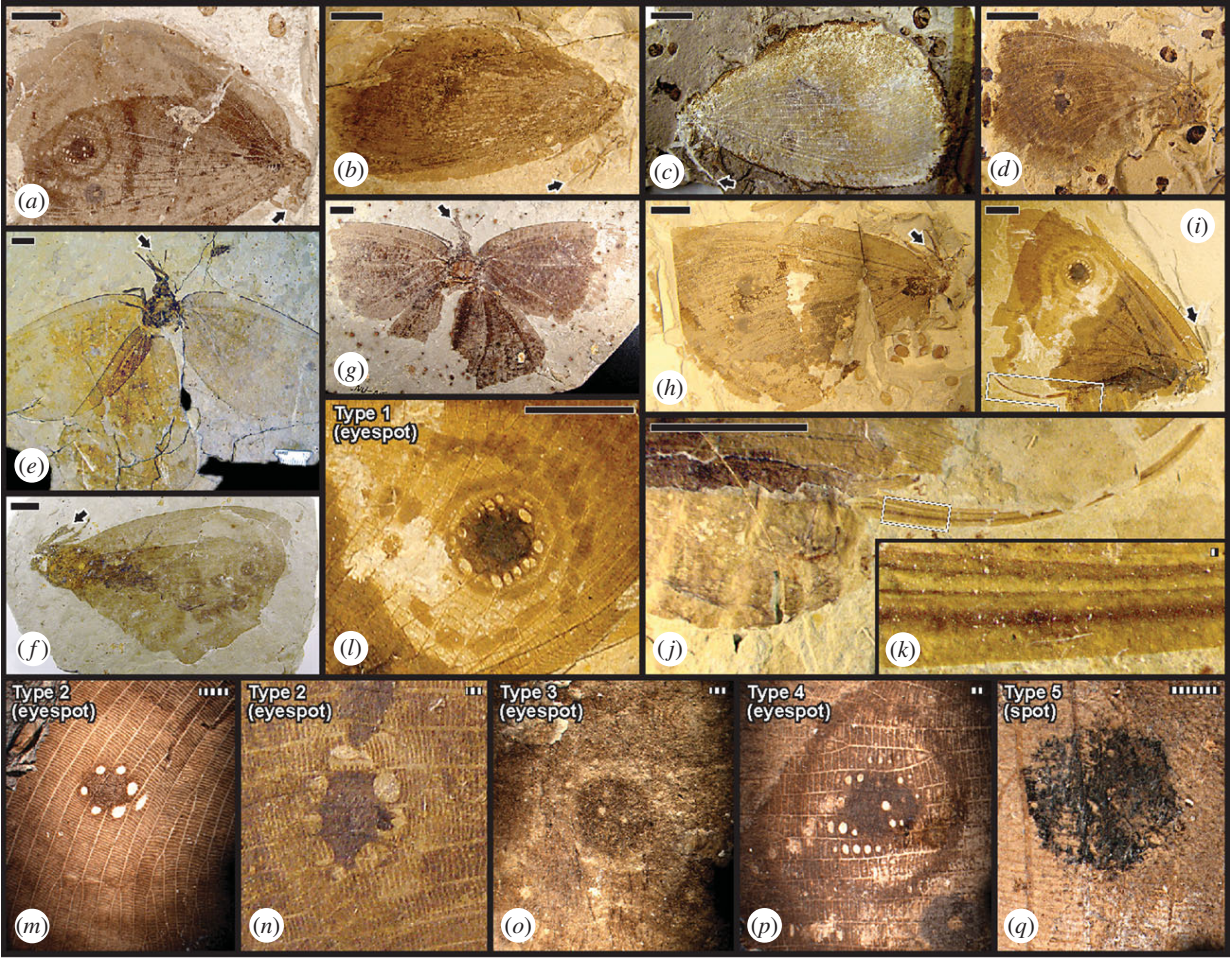
Kalligrammatid structural diversity. Specimens are from the late-Middle Jurassic Jiulongshan Fm. (JIU), China; Late Jurassic Karabastau Fm. (KAR), Kazakhstan; and mid-Early Cretaceous Yixian Fm. (YIX), China (electronic supplementary material, tables S2 and S3). At (*a*–*i*) are nine species showing general habitus [11]. Arrows indicate proboscis tips. (*a*) *Kalligramma circularia* (JIU); (*b*) *Affinigramma myrioneura* (JIU); (*c*) *A. myrioneura* (JIU); (*d*) *Kallihemerobius feroculus* (JIU); (*e*) *Oregramma aureolusa* (YIX); (*f*) *Ithigramma multinervia* (YIX); (*g*) *Abrigramma calophleba* (JIU); (*h*) *Kalligramma brachyrhyncha* (JIU); and (*i*) *Oregramma illecebrosa* (YIX). (*i*–*k*) Lateral views of ovipositor structure in *O. illecebrosa* above: (*i*) intact specimen; (*j*) complete ovipositor and posteriormost abdominal segments; and (*k*) lateral valve pairs. (*l*–*q*): five kalligrammatid wing eyespot and spot types detailed in figures 2 and 3; electronic supplementary material, figure S1. (*l*) Type 1 wing eyespot with two outer rings and *ca* 15 contiguous ocules surrounding a central pigmented disc (*O. illecebrosa*, YIX); (*m*) Type 2 wing eyespot with a single outer ring, light-hued inner area, and uninterrupted, pigmented central disc with surrounding, non-contiguous ocules (*Kallihemerobius almacellus*, JIU); (*n*) Type 2 eyespot similar to (M) (*Kallihemerobius feroculus*, JIU); (*o*) Type 3 wing eyespot with a light-hued circular area and a few, variably sized ocules in a darkly pigmented central disc (*Ithigramma multinervia*, YIX); (*p*) Type 4 wing eyespot contains a few ocules and others surrounding a pigmented central disc, a light-hued inner area and surrounding, dark outermost ring (*K. circularia*, JIU); and (*q*) Type 5 wing spot of a circular, pigmented central disc (*Kallihemerobius aciedentatus*, JIU). Scale bars: solid, 10 mm; striped, 1 mm.

Kalligrammatidae, or kalligrammatid lacewings (figure 1*a–i*), are an enigmatic, almost entirely Eurasian [11–13], mid-Mesozoic, holometabolous clade of large, robust-bodied Neuroptera (lacewings). Kalligrammatids had large wingspans, up to *ca* 160 mm [12], and are among the largest and most conspicuous of mid-Mesozoic insects (electronic supplementary material, table S1). Kalligrammatids were tentatively associated with seed plants [14–16], despite their almost unknown mouthpart and ovipositor structures [16]. Within Neuroptera, the Kalligrammatidae are included within Myrmeleontiformia [17–19], a major clade that encompasses extant antlions, owlflies, silky-winged lacewings (Psychopsidae), and spoon and thread-winged lacewings (Nemopteridae) [20,21]. The Nemopteridae share significant mouthpart and feeding similarities [21,22] with the Kalligrammatidae whereas the Psychopsidae possess similar wing features [16].

**Figure 2.**
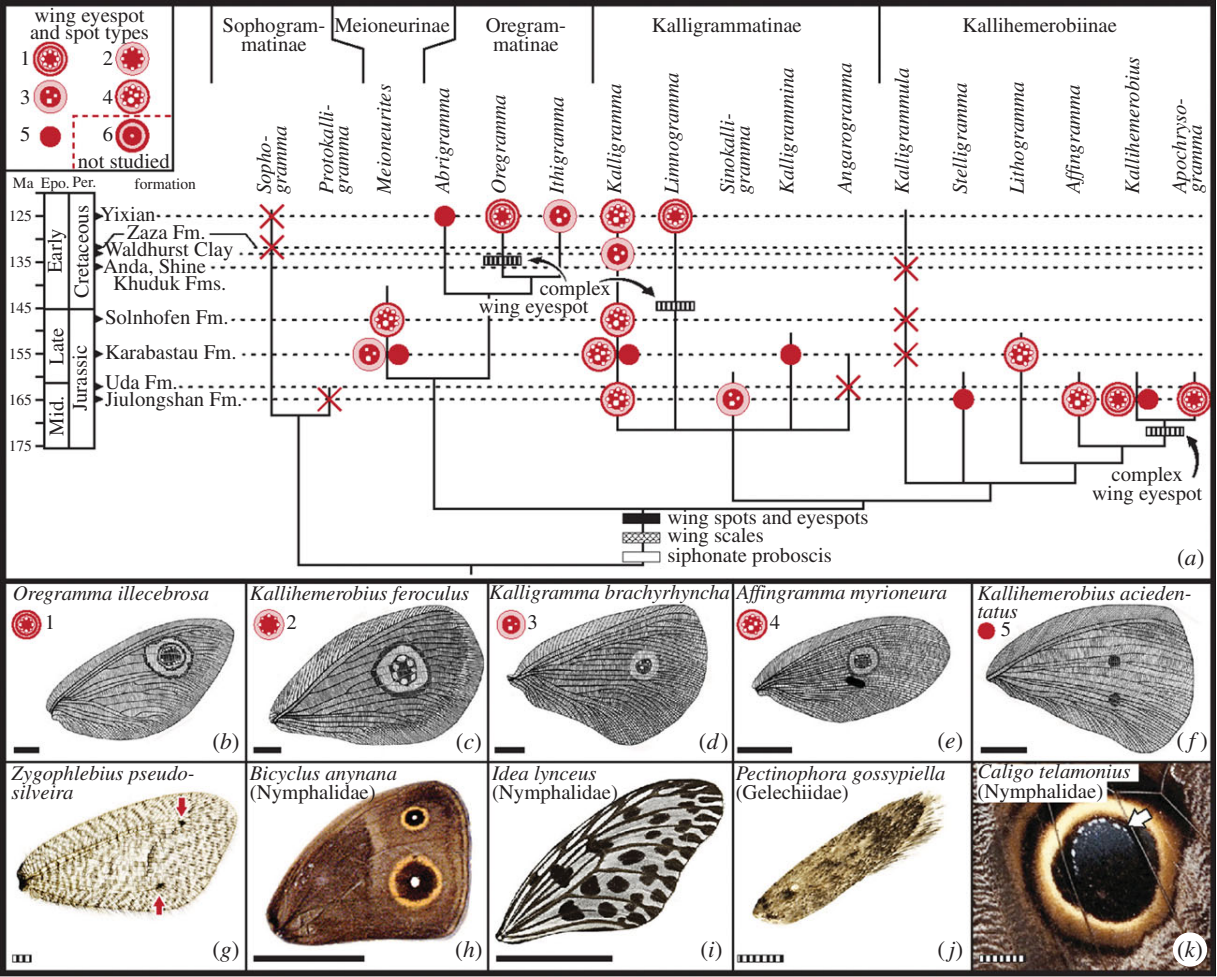
Phylogenetic context of wing spots and eyespots in mid-Mesozoic kalligrammatids, with comparisons to modern lepidopterans (electronic supplementary material, text S3). The best preserved fossil material was used for this analysis. (*a*) Most parsimonious tree of Kalligrammatidae phylogeny [11] (electronic supplementary material, table S2), with right forewing eyespot/spot condition mapped onto terminal clades and likely wing spot and eyespot origins. Wing eyespot and spot type symbols are at upper-left; crosses are eyespot/spot absences. (*b*–*g*) Examples of right forewings with wing eyespots or spots from mid-Mesozoic Kalligrammatidae (*b*–*f*), and modern Psychopsidae (*g*). These taxa correspond to a Type 1 eyespot (*b*), Type 2 eyespot (*c*), Type 3 eyespot (*d*), Type 4 eyespot (*e*) and two Type 5 double spots (*f*) matched by two spots in modern psychopsid (red arrows) in (*g*). Kalligrammatid wing eyespots and spots are compared to modern Lepidoptera in (*h*–*k*), of butterfly species with Type 6 eyespots (*h*) and multiple Type 5 spots (*i*); moth lacking wing spots or eyespots (*j*); and modern owl butterfly eyespot (*k*), showing pigmentation similar to Type 2 and 3 eyespots (*b*), indicated by arrow pointing to an ocule series and longitudinal wing vein. Scale bars: solid, 10 mm; striped, 1 mm.

All examined kalligrammatid material originated from fine-grained, often carbonaceous lake deposits in one Central Asian and two East Asian localities (figure 2*a*; electronic supplementary material, tables S2 and S3) [23–25]. The oldest deposit is Daohugou, of the Jiulongshan Formation, Inner Mongolia, from northeastern China. This deposit is radiometrically dated by ^40^K/^40^Ar at 164–165 Ma [26], a date supported by slightly younger isotopic dates from overlying volcanic deposits [26,27]. This date corresponds to the late Callovian of the latest Middle Jurassic, using a standard international timescale [28]. Diverse floras and the earliest known kalligrammatid lacewings occur at Daohugou [23]. Karatau, the middle deposit, is represented by the Mikhailovka and Aulie sites in the Chiment Region of eastern Kazakhstan. The date of this deposit, the Karabastau Formation [24], is uncertain within the Late Jurassic, but floras [29], insects [30] and stratigraphy [24] indicate a mid-Late Jurassic date, approximating 155 Ma. The youngest deposit, the Yixian Formation of Liaoning Province in northeastern China, consists of several sites separated in time and space. These sites encompass ^40^K/^40^Ar and ^87^Rb/^87^Sr dates ranging from 128.2 Ma low to 121.6 Ma high in the formation, with most material collected from the Jianshangou beds dated at *ca* 125 Ma [27,31], the date used in this report. Although contentious, Yixian dates are supported by a variety of palaeobiological evidence [27,32], buttressed by pollen studies [33] linked to a distinctive megaflora in the lower part of the unit [34]. Claims of a Late Jurassic age for Yixian fossils represent range extensions of Early Cretaceous lineages downward into the Late Jurassic [31]. The last known kalligrammatid lacewing occurs in the upper Crato Formation of northeastern Brazil, *ca* 120 Ma [13].

**Figure 3.**
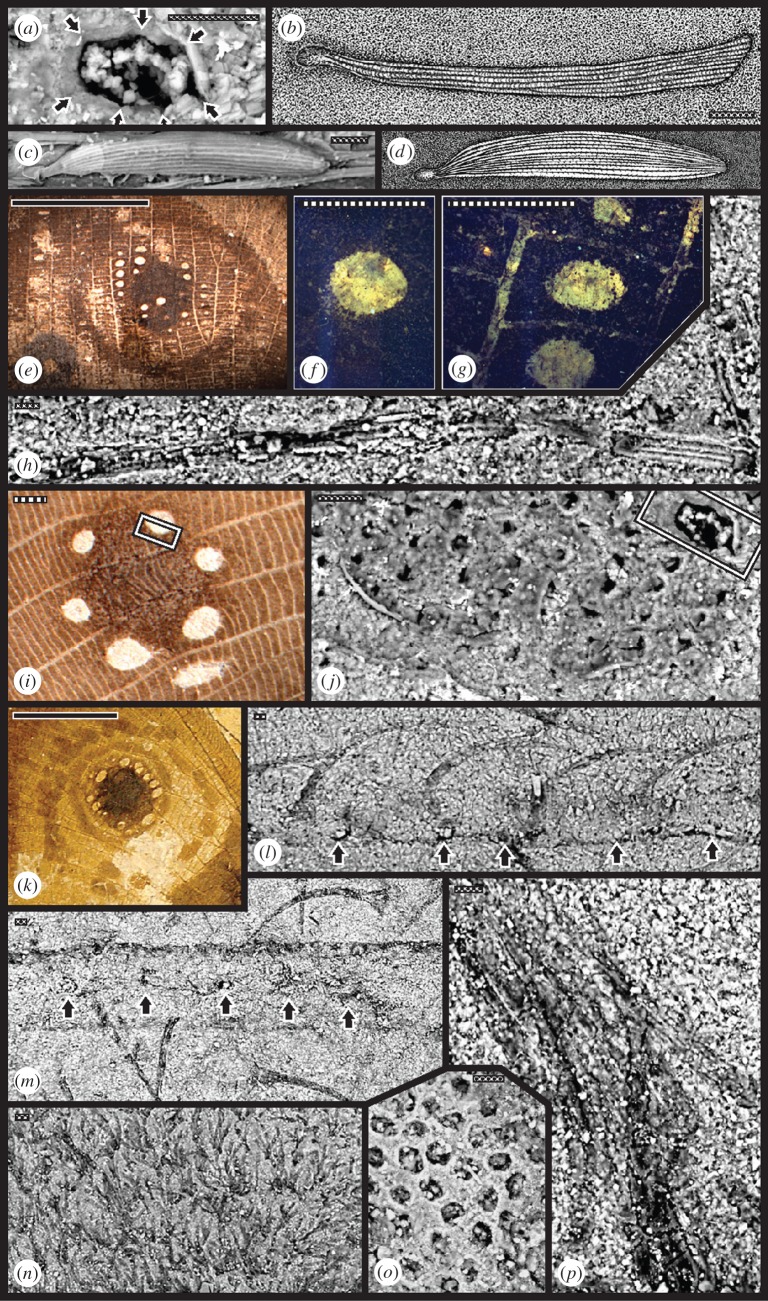
Microstructure of three kalligrammatid forewing eyespot types and their cuticular scales. (*a*) Kalligrammatid ellipsoidal wing-scale socket retains a broken scale base in cross-section of four lower (bottom arrows) and three upper (top arrows) ribs, enlarged from upper-right of (*j*). This socket type receives distinctive flat scales on major veins present elsewhere on the wing, depicted as an overlay drawing in (*b*), showing four longitudinal ribs basally and eight ribs terminally on *Kalligramma* sp. (JIU). For comparison of (*b*), at (*c*) and (*d*) is a foreleg scale of the modern neuropteran *Lomomyia squamosa* (Berothidae) (electronic supplementary material, text S2), in a SEM at left (*c*) and overlay drawing at right (*d*). (*e*–*h)* A Type 4 eyespot of *Kalligramma circularia* (JIU). (*e*) Light photograph showing eyespot pigmentation pattern, with epifluorescence microscopy revealing a differently pigmented ocule (*f*), and three additional ocules (*g*), each in a wing compartment surrounded by minor veins bearing flattened, four-ribbed scales, four shown in the SEM at (*h*). (*i*) Light photograph of a Type 2 eyespot of *Kallihemerobius almacellus* (JIU), showing seven whitish hued ocules surrounding a central pigmented disc, the boundary (template) shows smaller empty scale sockets in interveinal areas and occasional larger scale sockets on veins in the SEM at (*j*). Large wing-scale socket at upper-right enlarged at (*a*). (*k*–*n*) and (*p*) A light photograph of a Type 1 eyespot (*k*) from *Oregramma illecebrosa* (YIX), with dark pigmented central disc surrounded by whitish ocules and two dark outer rings. (*l*) SEM detail of four curved scales, each socketed on a longitudinal vein; black arrows indicate alternating sockets that lack scales. (*m*) Nearby scales. (*n*) Field of clumped scales on a wing region lacking veins and eyespots and a fascicle of eight, large, detached scales in (*p*), each displaying a ridged structure. Eyespot ocule at (*o*), from *Kallihemerobius aciedentatus* (JIU), shows a regular array of interveinal scale sockets, structurally distinct from central-disc pigmented regions, bearing scales socketed on major veins. See electronic supplementary material, table S2 for specimen data; scale bars: solid, 10 mm; striped, 1 mm; dotted, 10 µm.

Lake deposits such as the Jiulongshan, Karabastau and Yixian formations typically preserve plants and insects that reveal surface details [23,30,31]. Frequently, resolution of such features extends to colour patterns (figures 1*a*–*i*,*l*–*q* and 3*e*–*g*,*i*,*k*; electronic supplementary material, figure S2), gross (figure 1), to detailed mouthpart structure (electronic supplementary material, figures S1, S4 and S5), micromorphological details of wing and mouthpart scales (figure 3*a*,*b*,*h*,*j*,*l*–*p*; electronic supplementary material, figures S4 and S5), and reproductive plant features such as pollen (electronic supplementary material, figures S1*t*, S5*b* and S6*a*–*f*) and fructifications that reveal internal structures (electronic supplementary material, figure 6*g*–*i*) that extends previous studies [34–37].

## 2. Material and methods

The electronic supplementary material documents the general methodological approaches and specific experimental procedures used in six substudies that buttress our account of ultrastructure and morphology of Mesozoic kalligrammatid lacewings. These studies are (i) kalligrammatid mouthpart structure; (ii) an analysis of pigmentation within wing eyespots; (iii) geochemical analyses of opaque plugs trapped within the food canal of a tubular proboscis; (iv, v) two analyses on pollen occurring adjacent mouthpart contact surfaces; and (vi) taxonomic characterization of pollen in sedimentary matrices adjacent kalligrammatid specimens. We also provide documentation of kalligrammatid mouthpart morphology. The techniques contributing to these six substudies are briefly outlined below; details of instrumentation and equipment that were used, specific imaging procedures and the protocol for geochemical analyses are provided in the electronic supplementary material.

### (a) Specimen imaging

Light, epifluorescence and scanning electron microscopy (SEM) were used to closely examine a variety of kalligrammatid features from gross structure to micromorphology. Structures as miniscule and delicate as setae, wing scales, wing eyespot ocules and pollen grains were captured by microscopic imaging techniques, including the backscattering function linked to SEM imaging. Camera lucida drawings were made (electronic supplementary material, figure S1) to establish the most highly resolved scale available, and included shape, size, surface features and inter-element relationships of siphonate mouthpart structure.

### (b) Geochemical analyses

The heads, mouthparts, wing scales and eyespots of several specimens were intensely investigated by electron dispersion spectroscopy (EDS) linked to an environmental chamber SEM (electronic supplementary material, figure S2), also time-of-flight secondary ion mass spectrometry (ToF-SIMS, electronic supplementary material, figure S3) [38]. The latter technique produced intriguing results regarding eyespot pigmentation, and several EDS analyses characterized a structureless plug within the proboscis food canal of one specimen (electronic supplementary material, figure S4*e*–*j*). Pollen was detected adjacent vestigial but highly setose mandibulate mouthparts of a second specimen (electronic supplementary material, figure S4*a*–*d*). Two morphotypes of elongate cuticular scales were imaged from the mouthparts, particularly the maxillary palps, of another specimen using a variety of techniques that included SEM imaging (electronic supplementary material, figure S5). Wing eyespot pigmentation was detected by EDS by enhanced carbon concentrations that were intrinsic to the eyespot centre and absent from other regions such as the eyespot ocules, other body regions and adjacent rock matrix.

### (c) Pollen study

Most sedimentary matrices adjacent to the specimens that were acid macerated failed to preserve pollen, attributable to the oxidized condition of the encompassing rock. The matrix of one specimen, however, provided a well-preserved spectrum of pollen in macerated residues that were mounted on microscope slides for characterization. The resulting pollen was consistent not only with the known megaflora described from the same deposit but also provided common and rare entomophilous pollen taxa (electronic supplementary material, figure S6*a*–*f*).

## 3. Results

Recently, a comprehensive phylogenetic analyses of 30 wing (28 of 30), ovipositor and mouthpart characters for 17 kalligrammatid genera and four outgroups resulted in a single best-supported tree [11] (figure 2*a*). The phylogeny grouped the genera into five distinct clades, three of which are new subfamilies [11] (figure 2*a*; electronic supplementary material, table S1). The basalmost clade, Sophogrammatinae, represents the plesiomorphic kalligrammatid condition of mandibulate mouthparts and the absence of wing spots, eyespots, and scales. The four derived clades include Kalligrammatinae, consisting of the speciose *Kalligramma* and four related genera, and Kallihemerobiinae with six genera. Meioneurinae comprises the sole genus *Meioneurites* [16], which has a sister-group relationship to Oregrammatinae, the latter consisting of three genera, including probably the most derived genus, *Oregramma*. Higher-level relationships within Kalligrammatidae are: Sophogrammatinae + {[(Meioneurinae) + (Oregrammatinae)] + [(Kalligrammatinae) + (Kallihemerobiinae)]}.

In forewings, kalligrammatid eyespots and spots typically are deployed on the upper surface midway to two-thirds of the proximal-to-distal wing length, centred between two major branches of the radial vein system. Six distinctive types of forewing eyespots or spots occur on most species of the four derived kalligrammatid clades, occurrences previously known from some taxa [11,15], but not others [12]. The basalmost clade has no wing spots or eyespots (figure 2*a*), as do almost all modern neuropterans (figure 2*g*) [19]. There are four eyespot types, each consisting of distinctive, differentially pigmented rings surrounding a central pigmented disc with small, whitish, oval-shaped ocules (Types 1–4; figures 1*l*–*p* and 2*b*–*e*,*h*,*k*; electronic supplementary material, figure S2). In addition, there are taxa with two simple spots, consisting of a round, dark patch lacking concentric rings (Type 5; figures 1*q* and 2*f*). Eyespots of Type 6 were not mapped onto the phylogeny, as wing characters of one *Kalligramma* sp. were insufficiently preserved for inclusion in phylogenetic analyses. In Type 1 eyespots, a second ring of dark pigmentation occur relative to single ringed Types 2–4 and 6 (figures 1*l* and 2*b*).

Forewing eyespot and spot types (figure 1*l*–*q*) were mapped onto our best-supported tree (figure 2*a*), revealing major patterns. In all outgroup taxa and the basalmost kalligrammatid clade of Sophogrammatinae, eyespots and spots were absent. The evolution of spots and eyespots likely originated early within the kalligrammatid clade, in the sister lineage to Sophogrammatinae (figure 2*a*). The four kalligrammatid clades derived from this lineage exhibit a variety of spot and eyespot patterns and absences. The most complex eyespot type occurs late in three separate lineages, within Oregrammatinae (Type 1; figure 1*l*; electronic supplementary material, figure S2), Kallihemerobiinae and Kalligrammatinae (figure 2*a*), suggesting that these eyespots derive from simpler ones, a transition that likely happened multiple times. In addition, multiple simple spots were converted to single eyespots in several lineages. These patterns are similar to convergent changes conventionally proposed for nymphalid butterflies in modern Lepidoptera (figure 2*h*,*i*,*k*) [8,39,40]. Changes include transitions from moth taxa possessing monochromatic wings lacking differential pigmentation (figure 2*j*), to basal nymphalid taxa with simple repeated spots, such as *Idea lynceus* (figure 2*i*), to more elaborate and individualized eyespot patterns of *Bicyclus anynana* with multiple colour rings (figure 2*h*) [8,39]. The deployment of a spot of monochromatic pigment between two major veins in basal Kallihemerobiinae, Kalligrammatinae and Oregrammatinae (figure 2*a*,*f*) has convergently re-evolved in modern, distantly related Psychopsidae (figure 2*g*) and Nemopteridae [19].

Another point of convergence is the possible presence of melanin in wing eyespot centres as indicated by our EDS carbon (electronic supplementary material, figure S2) and ToF-SIMS (electronic supplementary material, figure S3) substudies. SEM examination of the eyespots using EDS revealed a significant increase in carbon content within black eyespot centres, whereas the central white pupil was completely devoid of carbon. In the ToF-SIMS analysis, the eumelanin presence was indicated by comparison of the spectrum from the dark eyespot pupil with the spectrum of a modern eumelanin standard. Owing to dissimilarities in the intensity of the organic peaks, similar to what has been found in other studies [41,42], the possibility of an alternative carbon source cannot be excluded. Unlike melanin preserved in many animals, where it occurs in rod-shaped specialized cells [43], insects lack such cells and melanin is diffused throughout the cuticle [44]. The relative abundances of carbon and the possible presence of melanin found in differently coloured regions of kalligrammatid eyespots could match the pigment distribution in many nymphalid eyespot patterns [39]. The muted response of carbon-rich material in kalligrammatid eyespots could mimic the nymphalid condition, as scales in an eyespot centre often are devoid of melanin and reflect all light wavelengths, appearing white [45], whereas black scales encircling the eyespot centre contain melanin [46].

Wing scales are another convergent feature occurring in Kalligrammatidae and modern Lepidoptera, although there are differences in detail. The basalmost clade, Sophogrammatinae, lacked wing scales, as do virtually all other modern, major neuropteran lineages (figure 3*c,d*). The four derived kalligrammatid clades bore two types of wing scales. The first type were large scales with a flattened, elongate-spatulate shape socketed on major veins and possessing three to four longitudinal ribs, increasing to six to eight ribs at the distal wider end (figure 3*a*,*b*,*j*,*p*; electronic supplementary material, figure S5*a*). The second scale type were small, short scales that were basally broad but tapered, bearing four or fewer longitudinal ribs, and originating from smaller sockets on areas between the major veins (figure 3*h*,*j*,*l*–*o*; electronic supplementary material, figure S3*d*). This distribution indicates wing scales originated de novo among early Kalligrammatidae, after separation from Sophogrammatinae (figure 2*a*). By comparison, in extant Lepidoptera, scales emerge predominantly from membrane surfaces and minor veins, but often are absent on major veins and larger cross-veins.

Mouthparts of kalligrammatid Neuroptera and papilionoid Lepidoptera offer another remarkable example of convergent evolution. Kalligrammatid mouthparts evolved from an ancestral mandibulate (chewing) state to a derived long-proboscid (siphoning) state in which maxillary elements were conjoined to form a tube (electronic supplementary material, figure S1). This parallels the evolution of the proboscis in glossate Lepidoptera, which also originated from mandible-bearing ancestors [47]. The kalligrammatid proboscis is present in all clades except basal Sophogrammatinae. Rudimentary, mandible-bearing mouthparts were retained in one long-proboscid specimen of Kallihemerobiinae (electronic supplementary material, figures S1*t*,*u* and S4), which bore a much-reduced labium and specialized mandibles, likely for pollen handling, indicated by adjacent pollen (electronic supplementary material, figure S1*t*). Rudimentary mandibles parallel that of the extant Nemopteridae (electronic supplementary material, figure S1*u*), probable sister-group of Kalligrammatidae [16], that currently have modified mandibulate mouthparts attached to an anterior prolongation of the head capsule for probing and nectaring flowers [9,19].

Many extinct and modern insects bear a long proboscis [9,14,36,48], but the proboscides of more derived kalligrammatids bear a special resemblance to those of Lepidoptera [47]. The kalligrammatid proboscis was long (8–20 mm), flexible, lacked stylets or other piercing structures, smooth or covered with surface hairs, bracketed by multisegmented maxillary palps, and its terminus typically rounded or truncate, resembling the end of a thick straw (electronic supplementary material, figure S1*b*,*e*)—all morphologies paralleling modern Lepidoptera [49]. In addition, kalligrammatid proboscides were longer and more robust, and thus differed from other coexisting, long-proboscid lineages, such as the shorter and more gracile, labellate pads borne by brachycerous flies [35,48], and analogous pseudolabellae of aneuretopsychine scorpionflies [36]. Suction forces were provided by one, perhaps two, sucking pumps located in the frontal head region (electronic supplementary material, figures S1 and S6*i*), mirroring those in Lepidoptera. The considerable mouthpart variation in kalligrammatids, especially of the proboscis, is comparable to modern Nymphalidae and other lepidopterans that probe for nectar and pollen at different floral depths and resistance [5,9,39]. Some kalligrammatid taxa bore thin and gracile proboscides (electronic supplementary material, figure S1*f*,*r*,*s*), and likely probed into narrow and shallow receptacles for ovular pollination drops and secretions from pollen organs [7,14]. By contrast, the robust and comparatively longer mouthparts of other kalligrammatid taxa (electronic supplementary material, figure S1*i*,*j*,*p*) were likely suited to probe larger, sturdier reproductive structures of Bennettitales, cycad-like plants contemporaneous with the Kalligrammatidae.

Three substudies (electronic supplementary material) explored the dietary range of kalligrammatid lacewings. The first examination targeted an opaque plug trapped within the food canal of a specimens' proboscis (electronic supplementary material, figure S4*e*–*j*), also seen under light microscopy (electronic supplementary material, figure S1*h*), indicating a bolus enriched in carbon and consistent with a diet of nectar-like fluids. A second assessment found pollen associated with the mouthparts of rudimentary mandibles in one specimen (electronic supplementary material, figures S1*t*,*u* and S4*a*–*d*). A third evaluation identified typical mid-Mesozoic, Eurasian pollen grains adjacent the maxillary palp base of another species (electronic supplementary material, figure S5). An additional substudy was a maceration of sedimentary matrix adjacent to several insect bodies, with pollen consistent with published megafloras from these localities (electronic supplementary material, figure S6*a*–*f*). These substudies document a similarity in feeding style and diet of kalligrammatid lacewings with extant butterflies.

Likely hosts for Kalligrammatidae include cycads (*Beania*), bennettitaleans (*Williamsonia, Weltrichia*) and caytonialeans (*Caytonia, Caytonianthus*). Members of the bennettitaleans and caytonialeans possessed the type of recessed ovules with tubular access that would receive long, probing proboscides of Kalligrammatidae [7,36,50–52]. Some Cheirolepidaceae possessed cone scales partially concealing deep funnels connected to ovules [35]. Early angiosperms from the Yixian Formation are delicate, aquatic, with small, nontubular flowers [34,53], unlikely hosts for Kalligrammatidae. Larger gymnospermous reproductive structures likely accommodated the more robust spectrum of kalligrammatid siphoning proboscides (electronic supplementary material, figure S1 and table S3).

Of all known Mesozoic gymnosperm groups, the bennettitalean family Williamsoniaceae most likely formed a close pollinator mutualism with the Kalligrammatidae. Six lines of evidence point to this inference. First, stoutly constructed and elongate kalligrammatid proboscides match the deeply placed fluids and pollen of bennettitaleans [7,50–52] (electronic supplementary material, figure S6*g*,*h*) better than other co-occurring proboscid-bearing taxa [13]. At least two Late Jurassic to Early Cretaceous Eurasian ovulate organs, *Williamsonia bryonyae*, and *W. minima*, had deep throats [50,52], and would have accommodated the longer proboscis lengths of kalligrammatid taxa, as would the Jiulongshan specimen (electronic supplementary material, figure S6*h*). Second, *Cycadopites* and other monosulcate pollen (electronic supplementary material, figure S6*c*) are present in the Jiulongshan [54], Karabastau [29,52] and Yixian [34] biotas, which also preserve diverse Kalligrammatidae [11] and williamsoniaceous male (*Weltrichia*) and female (*Williamsonia*) organs. Both taxa broadly coincide as fossils during a 60 million-year period of the mid-Mesozoic. Third, *Weltrichia* pollen organs (electronic supplementary material, figure S6*g*) bore secretory glands [50,51], interpreted as ‘nectaries’ [55], positioned below paired dehiscing pollen sacs along the inner surfaces of clasping bract-like structures [50,51,55]. Analogously, conspecific *Williamsonia* ovulate organs (electronic supplementary material, figure S6*h*) produced pollination droplets [35,52]. These nutritional rewards would have been lures for pollinator visits to male and female organs. Fourth, cheirolepidaceous and other conifer pollen occurred adjacent to the head and mouthparts on one kalligrammatid specimen (electronic supplementary material, figure S1*t*) [35], suggesting seed–plant pollen consumption and a predisposition for pollination [7], as pollen is often a supplemental protein source in modern pollinating insects [9,49]. Fifth, the presence of a curved, saw-like ovipositor (figure 1*i*–*k*), homologous and similarly shaped to that of the Dilaridae and used for inserting eggs into deep substrates [56], suggests that females sliced plant tissues for egg deposition and that their larvae consumed internal plant tissues, explaining insect galleries in williamsoniaceous tissues [35] and their expected occurrence in Early Cretaceous ambers [38]. Sixth, placement of *Weltrichia* and *Williamsonia* organs on separate parts of the same plant or on different conspecific plants [50,51], indicates an outcrossing reproductive strategy. For such functionally dioecious plants, wind may achieve moderate levels of fertilization, but insects are significantly more efficient [7].

## 4. Discussion and Conclusion

Several accounts [15,16]—some made nearly a century ago [57,58]—have opined on the superficial similarity of poorly preserved kalligrammatid lacewings with modern butterflies. Such analogies, however, were not based on detailed, ultrastructural, micro- and macromorphological, geochemical and palynological evidence. In this study, a broad array of evidence is marshalled to support structural convergence between mid-Mesozoic kalligrammatid lacewings and modern butterflies. This convergence extends to possible melanin presence, simpler spots to complex eyespots, wing scales, long-proboscid siphonate mouthparts, feeding style similarities, and associations with seed plants. These major convergences appeared twice in time and space, presumably under similar selective pressures.

Our data allow for inferences regarding the ecology of insect–predator antagonistic interactions. Similarities between kalligrammatid eyespots and butterfly eyespots lie in the use of concentric circles of pigmented cells to produce a conspicuous and contrasting display. This pattern was used either for predator intimidation or alternatively predator deflection to the wings away from the core body in extinct kalligrammatids, serving the same functions in butterflies [59,60]. Repeated evolution of eyespots from simpler multiple spots arose during the Middle Jurassic in Kalligrammatidae (figure 2*a*), closely paralleling Nymphalidae *ca* 110 Myr later [39]. An ecological explanation for why multiple wing spots were replaced by single wing eyespots in Kalligrammatidae may be the eyespot's larger and more effective startle or deterrent signal [61]. Eyespots likely were used to dissuade or deflect attacks by predators such as early birds or small theropod dinosaurs [60,61] or mantid insects [59].

Wing scales appeared in Middle Jurassic Kalligrammatidae and Early Palaeogene Lepidoptera. Previously, wing scales were not documented on other fossil or modern neuropterans. Our survey of NMNH Neuroptera (figure 3*c*,*d*) found a single occurrence of scales on the forewings of one genus of extant, unrelated Berothidae [62]. Although these scales have differences in branching and number of ribs compared to those of Kalligrammatidae (figure 3*b*), they likely are homologous. This indicates that wing-scale presence in the Kalligrammatidae and the absence in almost all other fossil and modern neuropterans may be due to changes in deployment of the gene regulatory network within wings, rather than independent origins of scales across Neuroptera.

There likely was an association between kalligrammatid lacewings and coexisting gymnosperm seed plants. Diverse evidence support this mid-Mesozoic association, including gymnosperm pollen grains occurring in proximity to the insects; mouthpart morphology designed for probing and fluid feeding; carbon-rich compounds in a kalligrammatid proboscis food tube; the contemporaneous existence of compatible gymnosperms bearing secretory tissues and other rewards in reproductive organs and elongate ovulate structures similar in tubular dimensions to probing kalligrammatid proboscides. This suite of structural features ended with the extinction of Kalligrammatidae and their plant hosts, coincident with the primary ecological expansion of angiosperms during the mid-Cretaceous at *ca* 125–90 Ma [7]. At this time, other functionally similar but anatomically analogous, long-proboscid mouthparts evolved in unrelated lineages, including Trichoptera (caddisflies) and Hymenoptera (wasps and bees) [9,14], which would have accessed angiosperm nectar [7,9,35].

Although understanding of the ecology in mid-Mesozoic insect clades is sparse [16], our study of Kalligrammatidae now establishes 20 genera and 51 valid species of plant-associated insects (figure 2*a*; electronic supplementary material, table S1). Kalligrammatidae are the most diverse and third major clade of recently recognized Eurasian, preangiospermous, long-proboscid insects [14], complementing brachycerous flies [35,48,63] and aneuretopsychine scorpionflies [36]. These multiple origins of long-proboscid insects [14] took place in a Jurassic world dominated by diverse gymnosperms with virtually no modern analogues [14,29,55,64]. Our data also suggest that if angiosperms antedated the mid-Early Cretaceous and were insect pollinated, they most likely harboured associations with mandibulate rather than long-proboscid insects, consistent with early angiosperm floral structure [7,19,35], and antedating the considerably more recent origin of distinctive tubular floral modifications that would accommodate long-proboscides [7,14]. Varied fossil data suggest that the mid-Cretaceous demise of many pre-existing gymnosperms led to extinction of their diverse insect associates [14,30,63–65], including Kalligrammatidae, during early angiosperm diversification. Intriguingly, this clade was replaced by ecologically convergent butterflies *ca* 60 Myr later.

## Supplementary Material

Data Supplement

## Data accessibility

Data forming the basis of this research and the details of analyses are available in the electronic supplementary material attached to this article.
