# Supplementary material for: The evolutionary convergence of mid-Mesozoic lacewings and Cenozoic butterflies
Source: Proc Biol Sci. 2016 Feb 10;283(1824):20152893. doi: 10.1098/rspb.2015.2893 (PMC4760178; doi:10.1098/rspb.2015.2893)
Supplement: Data Supplement [file rspb20152893supp1.docx]

**Data Supplement**

**The evolutionary convergence of mid-Mesozoic lacewings and Cenozoic butterflies**

**Conrad C. Labandeira, Qiang Yang, Jorge A. Santiago-Blay, Carol L. Hotton, Antónia Monteiro, Yongjie Wang, Yulia Goreva, ChungKun Shih, Sandra Siljeström, Tim R. Rose, David L. Dilcher, and Dong Ren^*^**

**Files in this Supplement: Materials and methods; locality and collector data; kalligrammatid diversity; specimen data tables; details of analytical techniques; light, epifluorescence and scanning electron microscope imaging; geochemical analyses such as electron dispersive spectroscopy and time-of-flight secondary ion mass spectrometry; and references.**

**Text S1.** Materials and Methods

**Methods S1.**Wing and eyespot studies using light, epifluorescence and environmental-chamber scanning electron microscopes.

**Methods S2.** Mouthpart studies using light, epifluorescence and environmental-chamber scanning electron microscopes

**Methods S3.** Geochemical analyses of mouthparts and wing eyespots

**Methods S4.** Pollen analyses using epifluorescence and environmental scanning electron microscopy, and matrix maceration techniques.

**Text S2.** Locality and Collector Data for Modern Taxa in Fig. 2

**Table S1.** Kalligrammatid Taxonomic Diversity

**Table S2.** Examined Specimens of Kalligrammatidae

**Table S3.** Kalligrammatid Mouthpart Measurement and Wing Eyespot/Spot Data

**Text S3.** Energy Dispersive Spectroscopy (EDS) Analysis of Wing Pigmentation

**Figure S2.** Light Microscopic and SEM Images with X-ray data for characterizing wing eyespots of a kalligrammatid specimen.

**Text S4.** Time-of-Flight Secondary Ion Mass Spectrometry (ToF-SIMS) Analysis of Wing Eyespot Pupal Composition

**Figure S3.** ToF-SIMS video and ion images and spectra of the eye spot of *Kallihemerobius feroculus*.

**Text S5.**Geochemical Analyses of Opaque Plugs within Food Canals.

**Figure S4.** Light microscopic and SEM Images with EDS data characterizing the mandibulate and siphonate mouthparts of two kalligrammatid specimens.

**Text S6.**Analyses of Pollen Occurring near Mouthpart Contact Surfaces.

**Figure S5**. Electron microprobe analysis of pollen at the palpal tips of *Meioneurites spectabilis* holotype (PIN-2784/1069), from the Late Jurassic of Karatau of eastern Kazakhstan

**Text S7.** Taxonomic Characterization of Pollen from Sedimentary Matrices adjacent Kalligrammatid Specimens.

**Figure S6.** Plant associations of kalligrammatids.

Figures S1–S6. Morphological Features of Kalligrammatid Gross Mouthparts, Mouthpart Elements, Wing Eyespots, Wing Eyespot Composition Pollen Associations, and Seed-Plant Relationships.

**Data Supplement References**

**Text S1. Materials and Methods**

An overview and specimen documentation of the experimental procedures is provided in the main text of this report.

**Methods S1.Wing and eyespot studies using light, epifluorescence and environmental-chamber scanning electron microscopes.** Light microscopy of wing eyespots and spots was imaged with an Olympus SZH stereomicroscope connected to an Olympus Q-Color 5^TM^ camera, followed by image processing with Image-Pro^®^ Plus software. Epifluorescence microscopy was done by an Axioskop compound microscope and the same imaging system as the stereomicroscopy. Higher resolution images were made on a Phillips XL-30 ESEM with a LaB6 electron source for uncoated specimens.

**Methods S2. Mouthpart studies using light, epifluorescence and environmental-chamber scanning electron microscopes.** For drawings of head and mouthparts, photo images were enlarged and stitched from an Olympus SZH stereomicroscope, from which overlay drawings were made by hand, inked, and then reduced to a standard scale for incorporation in figure 4. This technique allowed for retention and rendering of surface details such as setae, exoskeletal ornamentation and compound eye ommatidia. The same technique was used for wing and eyespot drawings in figure 2. General morphology was documented by an Olympus SZX12 stereomicroscope connected with an Olympus Q-Color 5^TM^ camera, with images processed by Image-Pro^®^ version 6.3 software. A Phillips XL-30 ESEM with a LaB6 electron source was used for detailed imaging for uncoated specimens. Additional imaging of micromorphology was done by a Zeiss epifluorescence Axioskop microscope connected with an Olympus Q-Color 3^TM^ camera whose images were processed by Q Capture Pro^TM^ version 6.0 software.

**Methods S3. Geochemical analyses of mouthparts and wing eyespots.** Kalligrammatid specimens were assessed for the geochemical composition of substances trapped in their mouthparts and wing eyespots. Of these, five specimens yielded informative geochemical and microstructural data from features such as wing eyespot pigmentation and scale patterns, proboscis food-tube contents, and the external surfaces of hairy mouthparts. The geochemical analyses were done by EDS on a FEI NOVA nanoSEM 600 FEG variable pressure scanning electron microscope equipped with a ThermoElectron EDS system. All EDS spectra included a significant contribution from the fossil matrix, and were purely qualitative. X-ray mapping showed the spatial distribution of Carbon within pigmented eyespots. Dark brown biological surfaces were targeted in some fossils, analyzed under high magnification, and shown to be rich in carbon. These surfaces often were associated with biological structures documented by a SEM. An examination of wing eyespot using time-of-flight secondary ion mass spectrometry (ToF-SIMS) was consistent with the presence of eumelanin, although the presence of an alternative carbon-based compound could not be ruled out in the centers of the eyespots. The ToF-SIMS analysis was performed on a ToF-SIMS IV (ION-TOF-GmbH) by rastering a 25 keV Bi_3_^+^ beam (pulsed current of 0.3 pA) over an area of ~ 300x300 µm for ~ 150 sec. The analyses were done in both positive and negative mode at high mass resolution (bunched mode: Δl ~ 3 µm, m⁄ Δ m ~ 3000-4000 at m⁄ z 30). As the samples acted as insulators, the surfaces were flooded with electrons for charge compensation during analysis. The standard of synthetic eumelanin was obtain from Sigma Aldrich (M8631).

**Methods S4.Pollen analyses using epifluorescence and environmental scanning electron microscopy, and matrix maceration techniques.** Heads, mouthparts and legs of kalligrammatid specimens were examined using a Zeiss Axioskop epifluorescence microscope outfitted with an EFGP longpass filter cube set (exciter HQ470/40x, dichroic Q495LP, emitter HQ500LP, Chroma filter set 41018). Fossil palynomorphs usually autofluoresced with a soft pink hue using this filter set. In addition, four specimens were examined with scanning electron microscopy using a Phillips XL-30 ESEM and a FEI NOVA nannoSEM-600 FEG variable pressure scanning electron microscope, imaged at 20 kV and 0.4 Torr.

To determine whether pollen was present but not observed due to failure to autofluoresce, the surrounding rock matrices of 12 specimens were macerated for pollen. The matrix samples of the other insect specimens were judged too oxidized to process. Sample processing by Global Geolab, Ltd., Canada, included acid maceration, heavy liquid separation and residue mounted in Elvacite or glycerine jelly, and staining with Bismarck Brown. Only four matrix samples yielded pollen, three from the Jiulongshan Fm., and one from the holotype of Meioneurites spectabilis from the Karabastau Fm. Palynomorphs in the M. spectabilis matrix sample were poorly preserved but autofluoresced brightly; this sample was heavily dominated by Classopollis pollen. The matrix from Kallihemerobius feroculus produced abundant, autofluorescent pollen. Matrix samples from two other Jiulongshan specimens contained abundant but very pale and poorly preserved palynomorphs that did not autofluoresce, disallowing confirmation of pollen on these insect specimens.

**Text S2.Locality and Collector Data for Modern Taxa in Figures 2 and 3**

The following are data associated with modern lacewing, butterfly and moth specimens in main text figure 2. These data are authorship updates and information recorded verbatim from the pin labels.

**Figure 2*g*.***Zygophlebis pseudosilveira* Oswald (Neuroptera: Psychopsidae). “South Africa, Tvi. Joogenoeg, Bewaarkool, near Chuniespoort 24.108 29.569 22.xi.1987 L.R. Minter. Paratype NMNH Entomology.”

**Figure 2*h*.***Bicyclus anynana* (Butler, 1879) (Lepidoptera: Nymphalidae). “Negzi Forest, Pemba Island Sept. 1963 coll. By A. Rydan. Kenya Natl. Mus. exchange.”

**Figure 2*i*.***Idealynceuslynceus* (Drury, 1773) (Nymphalidae). “Seechol Pen, Thailand Hugh Smith coll.”

**Figure 2*j*.***Pectinopora gossypiella* (Saunders, 1844) (Lepidoptera: Gelechiidae). “TEXAS: Nueces R., 5 mi SW Mathis, Aug. 12 1963, collectors Duckworth & Davis.”

**Figure 2*k*.***Caligo telamonius* Felder, 1862 (Lepidoptera: Nymphalidae. The yellow-fronted owl butterfly; Yale University Peabody Museum collection. “Specimen ID: 6208 Locality: Mexico: Veracruz: San Andres Tuxtla, Lago Encantada., leg. Raguso, R.A. 21.vii.1985.”

**Figure 3*c*,*d***. *Lomomyia squamosa* Carpenter 1940 (Neuroptera: Berothidae). “Mexico, Nayarit 20 m S. Tepic, VII-23-1963. P.J. Spangler.”

**Table S1. Kalligrammatid Taxonomic Diversity^1^**

*Taxon* *Distribution* *Geological Age and Formation* *Source*

**FAMILY KALLIGRAMMATIDAE**

**Subfamily Kalligrammatinae** Handlirsch, 1906 [1]

*Angarogramma* Ponomarenko, 1984 [2]

*A*. *incertum* Ponomarenko, 1984 Ulan Mayloulus, Russia Middle Jurassic, Uda Fm. [2]

*Kalligramma* Walther, 1904 [3]

*K. albifasciatum* Yang, Makarkin & Ren, 2014 Inner Mongolia, China Middle Jurassic, Jiulongshan Fm. [4]

*K*. *brachyrhyncha* Yang, Wang, Labandeira, Shih & Ren, 2014 Inner Mongolia, China Middle Jurassic, Jiulongshan Fm. [4]

*K*. *circularia* Yang, Wang, Labandeira, Shih & Ren, 2014 Inner Mongolia, China Middle Jurassic, Jiulongshan Fm. [4]

*K. delicatum* Liu, Khramov & Zhang 2015 Inner Mongolia, China Middle Jurassic, Jiulongshan Fm. [5]

*K. elegans* Yang, Makarkin & Ren, 2014 Inner Mongolia, China Middle Jurassic, Jiulongshan Fm. [6]

*K. flexuosum* Panfilov, 1968 Karatau, Kazakhstan Late Jurassic, Karabastau Fm. [7]

*K*. *haeckeli* Walther, 1904 Solnhofen, Germany Late Jurassic, Solnhofen Fm. [3]

*K*. *jurarchegonium* Zhang & Zhang, 2003 Liaoning, China Middle Jurassic, Haifanggou Fm. [8]

*K*. *liaoningense* Ren & Guo, 1996 Beipiao City, China Early Cretaceous, Yixian Fm. [9]

*K*. *multinerve* Panfilov, 1968 Karatau, Kazakhstan Late Jurassic, Karabastau Fm. [7]

*K*. *paradoxicum* Liu, Zheng, Zhang, Wang, Fang & Zhang, 2013 Inner Mongolia, China Middle Jurassic, Jiulongshan Fm. [3]

*K*. *roycrowsoni* Jarzembowski, 2001 Quarry Hill, United Kingdom Early Jurassic, Wadhurst Clay Fm. [10]

*K*. *sharovi* Panfilov, 1968 Karatau, Kazakhstan Late Jurassic, Karabastau Fm. [7]

*^1^Kalligramma* sp. Inner Mongolia, China Middle Jurassic, Jiulongshan Fm. [4]

*Kalligrammina* Panfilov, 1980 [11]

*K*. *areolate* Panfilov, 1980 Karatau, Kazakhstan Late Jurassic, Karabastau Fm. [11]

*Limnogramma* Ren, 2003 [12]

*L. hani* Makarkin, Ren & Yang, 2009 Inner Mongolia, China Middle Jurassic, Jiulongshan Fm. [13]

*L. mira* Ren, 2003 Beiopiao City, China Early Cretaceous, Yixian Fm. [12]

*L. mongolicum* Makarkin, Ren & Yang, 2009 Inner Mongolia, China Middle Jurassic, Jiulongshan Fm. [13]

*Sinokalligramma* Zhang, 2003 [14]

*S. jurassicum* Zhang, 2003 Inner Mongolia, China Middle Jurassic, Jiulongshan Fm. [14]

*Affinigramma* Yang, Wang, Labandeira, Shih & Ren, 2014 [4]

*A. myrioneura* Yang, Wang, Labandeira, Shih & Ren, 2014 Inner Mongolia, China Middle Jurassic, Jiulongshan Fm. [4]

*Taxon* *Distribution* *Geological Age and Formation* *Source*

**Subfamily Kallihemerobiinae** Ren & Engel, 2008 [15]

*Apochrysogramma* Yang, Makarkin & Ren, 2011 [16]

*A*. *rotundum* Yang, Makarkin & Ren, 2011 Inner Mongolia, China Middle Jurassic, Jiulongshan Fm. [16]

*Huiyingogramma* Liu, Zheng, Zhang, Wang, Fang & Zhang, 2013

*H*. *formosum* Liu, Zheng, Zhang, Wang, Fang & Zhang, 2013 Inner Mongolia, China Middle Jurassic, Jiulongshan Fm. [17]

*H*. *turutanovae* (Martynova, 1947) Karatau, Kazakhstan Late Jurassic, Karabastau Fm. [18]

*Kalligrammula* Martynova, 1947 [18]

*K*. *atra* Ponomarenko, 1992 Mongolia E. Cretaceous, Shine-Khuduk Fm. [19]

*K*. *karatavica* Martynova, 1947 Karatau, Kazakhstan Late Jurassic, Karabastau Fm. [18]

*K. karatensis* Liu, Khramov, Zhang & Jarzembowski, 2015 Inner Mongolia, China Middle Jurassic, Jiulongshan Fm. [20]

*K. lata* Liu, Khramov, Zhang & Jarzembowski, 2015 Inner Mongolia, China Middle Jurassic, Jiulongshan Fm. [20]

*K*. *senckenbergiana* Handlirsch, 1919 Solnhofen, Germany Late Jurassic, Solnhofen Fm. [21]

*Kallihemerobius* Ren & Oswald, 2002 [22]

*K*. *aciedentatus* Yang, Wang, Labandeira, Shih & Ren, 2014 Inner Mongolia, China Middle Jurassic, Jiulongshan Fm. [4]

*K*. *almacellus* Yang, Wang, Labandeira, Shih & Ren, 2014 Inner Mongolia, China Middle Jurassic, Jiulongshan Fm. [4]

*K*. *feroculus* Yang, Wang, Labandeira, Shih & Ren, 2014 Inner Mongolia, China Middle Jurassic, Jiulongshan Fm. [4]

*K*. *pleioneurus* Ren & Oswald, 2002 Inner Mongolia, China Middle Jurassic, Jiulongshan Fm. [22]

*Lithogramma* Panfilov, 1968 [7]

*L*. *oculatum* Panfilov, 1968 Karatau, Kazakhstan Late Jurassic, Karabastau Fm. [7]

*Stelligramma* Yang, Wang, Labandeira, Shih & Ren, 2014 [4]

*S. allochroma* Yang, Wang, Labandeira, Shih & Ren, 2014 Inner Mongolia, China Middle Jurassic, Jiulongshan Fm. [4]

^1^*Kallihemerobiinae* gen. indet. Inner Mongolia, China Middle Jurassic, Jiulongshan Fm. [4]

**Subfamily Meioneurinae**, Yang, Wang, Labandeira, Shih & Ren, 2014 [4]

*Meioneurites* Handlirsch, 1906 [1]

*M*. *schlosseri* Handlirsch, 1906 Solnhofen, Germany Late Jurassic, Solnhofen Fm. [1]

*M*. *spectabilis* Engel, 2005 Karatau, Kazakhstan Late Jurassic, Karabastau Fm. [23]

*M*. *villosus* Panfilov, 1968 Karatau, Kazakhstan Late Jurassic, Karabastau Fm. [7]

*Taxon* *Distribution* *Geological Age and Formation* *Source*

**Subfamily Oregrammatinae** Yang, Wang, Labandeira, Shih & Ren, 2014 [4]

*Abrigramma* Yang, Wang, Labandeira, Shih & Ren, 2014 [4]

*A. calophleba* Yang, Wang, Labandeira, Shih & Ren, 2014 Pingquan, Hebei, China Early Cretaceous, Yixian Fm. [4]

*Ithigramma*, Yang, Wang, Labandeira, Shih & Ren, 2014 [4]

*I. multinervia* Yang, Wang, Labandeira, Shih & Ren, 2014 Inner Mongolia, China Early Cretaceous, Yixian Fm. [4]

^1^*Ithigramma* sp. Inner Mongolia, China Early Cretaceous, Yixian Fm. [4]

*Oregramma* Ren, 2003 [12]

*O. aureolusa* Yang, Wang, Labandeira, Shih & Ren, 2014 Inner Mongolia, China Early Cretaceous, Yixian Fm. [4]

*O. gloriosa* Ren, 2003 Beipiao City, Liaoning, China Early Cretaceous, Yixian Fm. [12]

*O. illecebrosa* Yang, Wang, Labandeira, Shih & Ren, 2014 Beipiao City, Liaoning, China Early Cretaceous, Yixian Fm. [4]

^1^*Oregramma* sp. Inner Mongolia, China Early Cretaceous, Yixian Fm. [4]

**Subfamily Sophogrammatinae** Yang, Wang, Labandeira, Shih & Ren, 2014 S4

*Protokalligramma* Yang, Makarkin& Ren, 2011 [16]

*P. bifasciatum* Yang, Makarkin& Ren, 2011 Inner Mongolia, China Middle Jurassic, Jiulongshan Fm. [16]

*Sophogramma* Ren & Guo [9]

*S. eucallum* Ren & Guo, 1996 Beipiao City, Liaoning, China Early Cretaceous, Yixian Fm. [9]

*S*. *lii* Yang, Zhao & Ren, 2009 Beipiao City, Liaoning, China Early Cretaceous, Yixian Fm. [24]

*S*. *papilionacea* Ren & Guo, 1996 Beipiao City, Liaoning, China Early Cretaceous, Yixian Fm. [9]

*S*. *pingquanica* Yang, Wang, Labandeira, Shih & Ren, 2014 Pingquan, Hebei, China Early Cretaceous, Yixian Fm. [7]

*S*. *plecophlebia* Ren & Guo, 1996 Beipiao City, Liaoning, China Early Cretaceous, Yixian Fm. [9]

^1^*Sophogramma* sp. Baissa, Russia Early Cretaceous, Zaza Fm. [4]

Uncertain assignment

*Palparites deichmuelleri* Handlirsch, 1906 Solnhofen, Germany Late Jurassic, Solnhofen Fm. [1]

*Makarkinia kerneri* Bechly & Makarkin, 2016 Araripe Basin, Brazil Lower Cretaceous, Crato Fm. [25]

^1^Lower-level taxon undefined (5 species). SUMMARYFOR KALLIGRAMMATIDAE: 5 Subfamilies, 20 genera, 51 valid species.

**Table S2. Examined Specimens of Kalligrammatidae**

*Specimen* *Genus and species* *Authors* *Locality* *Age*

1. CNU-NEU-NN-2009-006P/C *Affinigramma myrioneura* Yang, Wang, Labandeira, Shih & Ren Daohugou Middle Jurassic

(Bajocian–Callovian)

2. CNU-NEU-NN-2009-007P/C *Affinigramma myrioneura* Yang, Wang, Labandeira, Shih & Ren Daohugou Middle Jurassic

(Bajocian–Callovian)

3. CNU-NEU-NN-2010-008P/C *Kallihemerobius aciedentatus* Yang, Wang, Labandeira, Shih & Ren Daohugou Middle Jurassic

(Bajocian–Callovian)

4. CNU-NEU-NN-2009-050P/C *Kallihemerobius almacellus* Yang, Wang, Labandeira, Shih & Ren Daohugou Middle Jurassic

(Bajocian–Callovian)

5. CNU-NEU-NN-2010-013P/C *Kallihemerobius feroculus* Yang, Wang, Labandeira, Shih & Ren Daohugou Middle Jurassic

Bajocian–Callovian)

6. CNU-NEU-NN-2010-012P/C *Stelligramma allochroma* Yang, Wang, Labandeira, Shih & Ren Daohugou Middle Jurassic

(Bajocian–Callovian)

7. CNU-NEU-NN-2009-033 Kallihemerobiinae gen. & sp. indet. Yang, Wang, Labandeira, Shih & Ren Daohugou Middle Jurassic

(Bajocian–Callovian)

8. CNU-NEU-NN-2009-030P/C *Kalligramma brachyrhyncha* Yang, Wang, Labandeira, Shih & Ren Daohugou Middle Jurassic

(Bajocian–Callovian)

9. CNU-NEU-NN-2010-015P/C *Kalligramma circularia* Yang, Wang, Labandeira, Shih & Ren Daohugou Middle Jurassic

(Bajocian–Callovian)

10. CNU-NEU-NN-2010-003 *Kalligramma circularia* Yang, Wang, Labandeira, Shih & Ren Daohugou Middle Jurassic

(Bajocian–Callovian)

11. CNU-NEU-NN-2010-011 *Kalligramma circularia* Yang, Wang, Labandeira, Shih & Ren Daohugou Middle Jurassic

(Bajocian–Callovian)

12. CNU-NEU-NN-2010-010P/C *Kalligramma* sp. Yang, Wang, Labandeira, Shih & Ren Daohugou Middle Jurassic

(Bajocian–Callovian)

13. CNU-NEU-HP-2009-001P/C *Abrigramma calophleba* Yang, Wang, Labandeira, Shih & Ren Pingquan Early Cretaceous

(Barremian)

14. CNU-NEU-NN-2009-034P/C *Ithigramma multinervia* Yang, Wang, Labandeira, Shih & Ren Liutiaogou Early Cretaceous

(Barremian)

15. CNU-NEU-NN-2010-016P/C *Ithigramma* sp. Yang, Wang, Labandeira, Shih & Ren Liutiaogou Early Cretaceous

(Barremian)

16. CNU-NEU-NN-2009-032P/C *Oregramma aureolusa* Yang, Wang, Labandeira, Shih & Ren Liutiaogou Early Cretaceous

(Barremian)

17. CNU-NEU-LB-2009-031P/C *Oregramma illecebrosa* Yang, Wang, Labandeira, Shih & Ren Huangbanjigou Early Cretaceous

(Barremian)

*Specimen* *Genus and species* *Authors* *Locality* *Age*

_________________________________________________________________________________________________________________________________

18. CNU-NEU-NN-2010-014P/C *Oregramma* sp. Yang, Wang, Labandeira, Shih & Ren Liutiaogou Early Cretaceous

(Barremian)

19. CNU-NEU-NN-2010-009P/C *Sophogramma pingquanica* Yang, Wang, Labandeira, Shih & Ren Pingquan Early Cretaceous

(Barremian)

20. PIN-2784/1069 *Meioneurites spectabilis* Engel Karatau Late Jurassic

(Kimmeridgian)

**Table S3. Kalligrammatid Mouthpart Measurement and Wing Eyespot/Spot Data**

*Text and* **------------------------***Proboscis***-------------------------** *Proboscis Wing spot*

*Species and* *Locality* *Data Supp.* *Length* *Width* *Food canal* *to palpal Antennal or eyespot*

*specimen number* *and age* *figures* *(mm)* *(mm)* *diam. (mm)* *Terminus* *Surface* *length ratio* *segments* *category*

*Affinigramma myrioneura* Daohugou 1*c*;2*e*; 13.9 0.8 0.4 truncate smooth proboscis < 30 Type 4

CNU-NEU-NN-2009-006P/C Middle Jurassic S1*r* –smooth > palps (eyespot)

*Affinigramma myrioneura* Daohugou 1*b*;2*e*; 14.0 0.6 0.3 truncate smooth proboscis < 30 Type 4

CNU-NEU-NN-2009-007P/C Middle Jurassic S1*f*,*s* –smooth > palps (eyespot)

*Kallihemerobius aciedentatus* Daohugou 1*q*;2*f*;3*o*; 9.8 0.9 0.2 rounded minutely proboscis 30–50 Type 5

CNU-NEU-NN-2010-008P/C Middle Jurassic S1*c*,*q* ridged = palps (spot)

*Kallihemerobius almacellus* Daohugou 1*m*;3,*l*,*j*; 9.1 0.7 0.2 truncate smooth proboscis ? Type 2

CNU-NEU-NN-2009-050P/C Middle Jurassic S1*d*,*m* smooth = palps (eyespot)

*Kallihemerobius feroculus* Daohugou 1*d*;2*c*; ― 1.4 0.6 ? smooth ? ? Type 2

CNU-NEU-NN-2010-013P/C Middle Jurassic *S1*t*;S3*a* (eyespot)

*Stelligramma allochroma* Daohugou ― ----------------------------mouthparts not preserved------------------------------ Type 5

CNU-NEU-NN-2010-012P/C Middle Jurassic (spot)

Kallihemerobiinae gen. indet. Daohugou S1*p*;4*g* 10.3 1.3 0.4 rounded smooth proboscis 30–50 ?

CNU-NEU-NN-2009-033 Middle Jurassic > palps

*Kalligramma brachyrhyncha* Daohugou 1*h*;2*d*;S1*h*, 11.2 1.0 0.5 truncate smooth proboscis ? Type 4

CNU-NEU-NN-2009-030P/C Middle Jurassic *w*;S3*e*-*j* –smooth > palps (eyespot)

*Kalligramma circularia* Daohugou ― ----------------------------mouthparts not preserved------------------------------ Type 4

CNU-NEU-NN-2010-015P/C Middle Jurassic (eyespot)

*Kalligramma circularia* Daohugou 1*a*,*p*;3*e*- 8.5 0.7 ? rounded minutely proboscis 30–50 Type 4

CNU-NEU-NN-2010-003 Middle Jurassic *h*;S1*o*; ridged > palps (eyespot)

S1*a*-*d*

*Kalligramma circularia* Daohugou S1*v* 5.4 0.5 ? rounded smooth proboscis ? Type 4

CNU-NEU-NN-2010-011 Middle Jurassic palps (eyespot)

*Kalligramma* sp. Daohugou S1*x* >10.0 1.7 ? ? minutely ? ? Type 6

CNU-NEU-NN-2010-010P/C Middle Jurassic ridged (eyespot)

*Abrigramma* *calophleba* Hebei–Pingquan 1*g*,S1*a*,*g* 10.7 2.3 0.9 rounded setate proboscis 30–50 Type 5

CNU-NEU-HP-2009-001P/C Early Cretaceous > palps (spot)

*Ithigramma multinervia* Liutiaogou 1*o*;S1*n* 9.8 1.1 ? ? setate proboscis ? Type 3

CNU-NEU-NN-2009-034P/C Early Cretaceous = palps (eyespot)

*Ithigramma* sp. Liutiaogou S1*k* >9.4 0.7 ? truncate smooth proboscis ? Type 3

CNU-NEU-NN-2010-016P/C Early Cretaceous –setate < palps (eyespot)

*Oregramma aureolusa* Liutiaogou 1*e*;S1*i* >18.0 1.1 0.9? ? smooth ? > 50 Type 1

CNU-NEU-NN-2009-032P/C Early Cretaceous (eyespot)

*Oregramma illecebrosa* Liaoning–Beipiao 1*i*-*l*;2*b*;3*k*- 16.1 0.8 0.3 subterm. setate proboscis 30–50 Type 1

CNU-NEU-LB-2009-031P/C Early Cretaceous *n*,*p*;4*b*-*e*; constrict. >palps (eyespot)

S1*l*

*Oregramma* sp. Liutiaogou S1*j* 7.6 1.0 ? ? minutely proboscis ? Type 1

CNU-NEU-NN-2010-014P/C Early Cretaceous ridged > palps (eyespot)

*Sophogramma pingquanica* Hebei–Pingquan ― -----------------------mouthparts not preserved----------------------- > 50 absent

CNU-NEU-HP-2010-009P/C Early Cretaceous

*Meioneurites spectabilis* Karatau, Late S1*l*;S4*a*- ? ? ? ? ? proboscis > 50 absent

PIN-2784/1069 Jurassic *d* < palps

*****Ventral aspect, with labium and monocondylously articulating, palmate mandibles present associated with pollen; proboscis incomplete.

**Text S3.Energy Dispersive Spectroscopy (EDS) Analysis of Wing Pigmentation (Figure S2, *below*)**

To explore patterns of wing pigmentation, a Type 4 wing eyespot of *Kalligramma circularia* from the Middle Jurassic was examined (see figure S2). One targeted region within this eyespot is illustrated in figure S2*c*, *d*). Energy dispersive spectroscopy (EDS) backscattered image revealed a blotchy pigmented portion of an eyespot with a more uniform portion of a central white ocule (figure S2*c*, right side). The chemical distribution of EDS Carbon (red) and Silicon (blue) X-ray K α maps of the identical quadrat is superimposed, showing enrichment of carbon in the pigmented area and its comparative absence in the ocule (figure S2*d*). This finding indicates that the conspicuous pigmentation pattern could possibly be due to melanin within the dark area of an eyespot, as melanin is a chemically stable molecule difficult to solubilize and enriched in carboxylic groups [26]. Carbon enrichment is detected in in the wing scales as well, but with far lesser extent than within dark eyespot, indicating that the pigmented and non-pigmented patterns are due predominantly to wing membrane colors, rather than to differently colored wing scales, also seen in some Papilionoidea [27].

**Text S4. Time-of-Flight Secondary Ion Mass Spectrometry (ToF-SIMS) Analysis of Wing Eyespot Pupal Composition (Figure S3, below)**

The eye spot of *Kallihemerobius feroculus* (Figure 1*d*, *n*) was analyzed with time-of-flight secondary ion mass spectrometry (ToF-SIMS) to seek the presence of eumelanin. The ToF-SIMS ion images (figure S3) show that peaks that are commonly assigned to eumelanin (*m/z* 50.00, 66.00, 73.01, 74.01, 97.01, 98.00, 121.01, 122.01, 145.00 and 146.000 [28] principally are localized at the darker area of the eyespot while peaks which can be assigned to the mineral matrix SiO_x_ (*m/z* 59.97 and 75.96) and SiO_x_H (*m/z* 76.97) are localized at the lighter area of the eyespot. There also are increased intensities of the peaks assigned to eumelanin in the eyespot spectrum compared to spectra from the rest of the fossil and the surrounding matrix.

There are clear similarities between the spectrum from the eyespot and that of the eumelanin standard (synthetic Sigma-Aldrich M8631) (figure S3), such as the presence of all peaks commonly assigned to melanin [28]. However, there also are dissimilarities between the two spectra, in addition to the difference caused by the presence of inorganic ions. For example, the eumelanin peaks representing the C_x_N series (*m/z* 74, 98, 122 and 146) seem to be depleted compared to peaks representing the C_x_ and C_x_H (*m/z* 72, 73, 96, 97, 120, 121, 144 and 145) series [28] in the spectrum of the eyespot. Similar differences in intensity can be observed in published spectra of fossilized eumelanin in other studies [29,30]. Due to the differences in the spectra of the fossil and standard, and the lack of melanosomes in the fossil, the organic signal in the spectrum of the eyespot can only tentatively be assigned to eumelanin, as another fossilized carbon source cannot be completely ruled out.

**Text S5: Geochemical Analyses of Opaque Plugs within Food Canals (Fig. S3*e*–*j*, *below*).**

Geochemical analyses targeting trapped, opaque plugs within food canals (figures S3*e*–*j*), also seen under light microscopy (figure S3*e*), indicate that some kalligrammatids consumed carbon-rich compounds consistent with a diet of nectar-like fluids found in Lepidoptera. In particular, the proboscis food canal of *Kalligramma brachyrhyncha* (figure S3*e*) was enlarged under an SEM to produce a backscattered image of the food canal, surrounded on each side by proboscis maxillary elements (figure S3*f*). Chemical analyses of trapped plug of organic material (figure S3*g*) indicated elevated carbon levels consistent with carbohydrates, such as pollen drops [31], but lacked evidence for iron, which would indicate blood feeding (figure S3*h*). Other proboscis-associated structures included a linear pattern of socketed scale placement (figure S3*i*), and enigmatic hair-like structures (figure S3*j*).

**Text S6: Analyses of Pollen Occurring near Mouthpart Contact Surfaces (Figs.S3*a*–*d*, S4, *below*).**

Several individual grains of pollen and spores were observed on the head area of *Kallihemerobius feroculus* (Fig. S3*a*–*d*). One bisaccate grain was noted very near a palmate-shaped mandible (figures S1*t* inset, S3*a*). Further examination of the right mandible (figure S3*a*) revealed a distinctive surface pattern of dense, overlapping setae and moderate amounts of carbon, which may indicate pollen feeding. The hirsute condition of the mandible surface contrasts with sparsely deployed setae elsewhere on the head underside, a feature similar to extant, flower visiting, African Nemopteridae (figure S1*u*) [32,33].Unfortunately, the head capsule was poorly preserved and the observed pollen and other grains may have been associated with the matrix. Pollen was observed elsewhere on the matrix surface of this specimen under epifluorescence.

Scattered grains and small clumps of pollen were observed on the legs and mouthparts of Late Jurassic *Meioneurites spectabilis*, confirmed by SEM to be *Classopollis* pollen of cheirolepidaceous conifers (figures 4*b*,*e*), and also in the matrix surrounding this specimen. Notably, *Classopollis* grains occur in the guts of several taxa of pollen feeding insects at the same site [34], consistent with our EDS analysis. SEM micrographs revealed the presence of large, elongate, ribbed scales oriented parallel to the palpal axis (figure S4*a*). These scales occasionally bore *Classopollis* cf. *annulatus* pollen tetrads (figure S4*b*), lodged among a dark, cracked, surface of palpal scales (figures S4*c*, *d*). These dense palpal scales were common and considerably larger than the nonribbed, smaller setae occurring elsewhere on the specimen’s mouthparts; they may represent specialized brushes for capturing pollen [35]. This specimen is the best evidence to date of a pollen feeding relationship between Kalligrammatidae and seed plants.

**Text S7: Taxonomic Characterization of Pollen from Sedimentary Matrices adjacent Kalligrammatid Specimens (Figs. 3*a*–*f*, S6, *below*).**

Pollen was rare in the matrix sediment of most of the specimens under investigation, likely a consequence of poor preservation in oxygen-rich source sediments or postdepositional oxidation of sediments. Only matrix surrounding the Kallihemerobius feroculus specimen (figures 1d; S3a–d) from the Jiulongshan Formation produced reasonably well preserved pollen, congruent with the reported megafloral composition of major plant groups present [36–39]. The observed constituents were not particularly age diagnostic, but consistent with a late Middle Jurassic date [40]. The flora was dominated by conifers, especially bisaccate pollen representing the Pinaceae or its antecedents. Classopollis pollen (figures S5b; S6e) had a subdominant position. Other conifers, such as the Araucariaceae, were rare. Possible candidates for seed-plant hosts, such as the Caytoniaceae (figure S6d), were very rare; as were the Cycadales, Bennettitales (figures S6g, h), Ginkgoales and Czekanowskiales, collectively represented by smooth or minutely ornamented, monosulcate pollen (figures S6a, c). Several grains of Eucommiidites, an extinct seed plant group perhaps related to Gnetales or Bennettitales [41], also were observed. Spores were uncommon in the matrix and included representatives of three fern families (figure S6f) and club-mosses. Pollen and megafloral identifications contain typical mid Mesozoic representatives of lycophytes, sphenophytes, ferns, and seed plants such as conifers, cycads, bennettitaleans, caytonialeans, ginkgoaleans and czekanowskialeans [36–38]. The Lower Cretaceous Yixian Formation also possessed rare angiosperms (Archaefructus, Hyrcantha) [38,39], gnetaleans (Ephedrites, Gurvanella), and other seed plants of uncertain affiliation (Problematospermum) [38].

* * * * * * *

Figures S1–S6: Morphological Features of Kalligrammatid Gross Mouthparts, Mouthpart Elements, Wing Eyespots, Wing Eyespot Composition. Pollen Associations, and Seed-Plant Relationships.

**
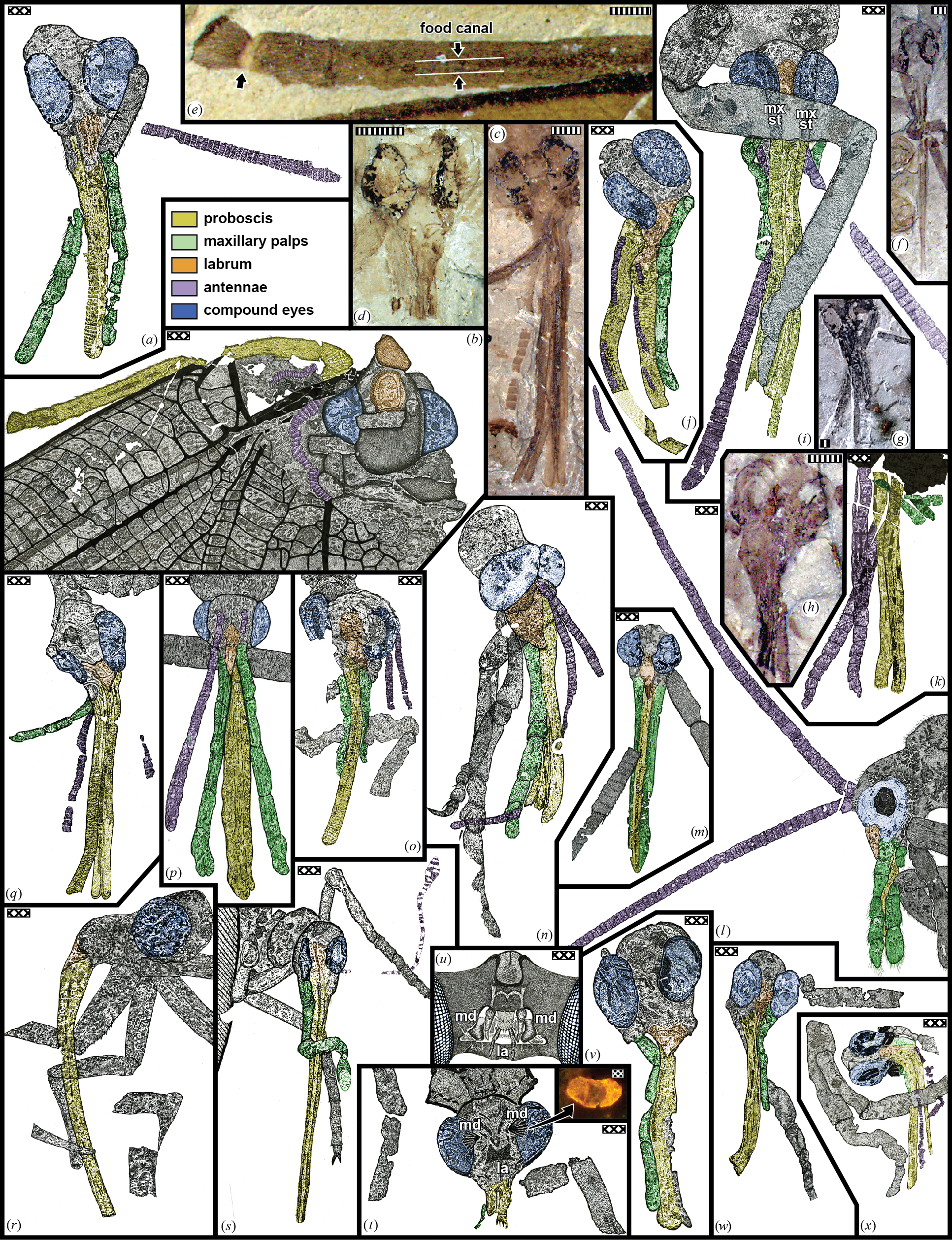
**

**Figure S1. Gross mouthpart diversity and proboscis variation in kalligrammatid lacewings from the mid Mesozoic of Eastern Asia (*previous page*).** Drawings and digital images of kalligrammatid taxa from the late Middle Jurassic (Jiulongshan Fm., 165 Ma, JIU) of northeastern China (*c*, *d*, *f*, *h*, *m*, *o*–*t*, *v*–*x*), mid Late Jurassic (Karabastau Fm., 155 Ma, KAR) of Kazakhstan (*l*), and mid Early Cretaceous (Yixian Fm., 125 Ma, YIX) of northeastern China (*a*, *b*, *e*, *g*, *i*–*k*, *n*). All overlay drawings are standardized to a scale of 5 mm (double diamond scale bar) to show size relationships; head and mouthpart elements are color identified to legend at upper left. (***a***, ***g***) *Abrigramma calophleba* (YIX, dorsal view); (***b*, *e***) *Oregramma illecebrosa* (YIX, dorsal view), with food canal and subterminal constriction (arrow); (***c***, ***q***) *Kallihemerobius aciedentatus* (JIU, dorsal view); (***d***, ***m***) *Kallihemerobius almacellus* (JIU, dorsal view); (***f***, ***s***) *Affinigramma myrioneura* (Jiulongshan, dorsal view); (***h***, ***w***) *Kalligramma brachyrhyncha* (Jiulongshan, dorsal view); (***i***) *Oregramma aureolusa* (YIX, ventral view), with prominent maxillary stipites (**mxst**); (***j***) *Oregramma* sp. (YIX, oblique lateral view); (***k***) *Ithigramma* sp. (YIX, lateral view); (***l***) *Meioneurites spectabilis* (KAR, left lateral view); (***n***) *Ithigramma multinervia* (YIX, right oblique view); (***o***) *Kalligramma circularia* (JIU, right lateral view); (***p***) Kallihemerobiinae gen. et sp. indet. (JIU, dorsal view); (***r***) *Affinigramma myrioneura* (JIU, left lateral view); (***s***) *Affinigramma myrioneura* (JIU, frontal view); (***t***) *Kallihemerobius feroculus* (JIU, ventral view), with modified mandibles (**md**) adjacent the labial plate (**la**) and associated bisaccate pollen, probably Pinaceae, near the left mandible; (***u***) Ventral view of mandibles and labial plate of an extant, pollinating South African species of Nemopteridae [32], for comparison to (T); (***v***) Another specimen of *Kalligramma circularia* (JIU, dorsal view); (***x***) *Kalligramma* sp. (JIU, dorsal view). Scale bars: striped, 1 mm; dotted, 10 mm.


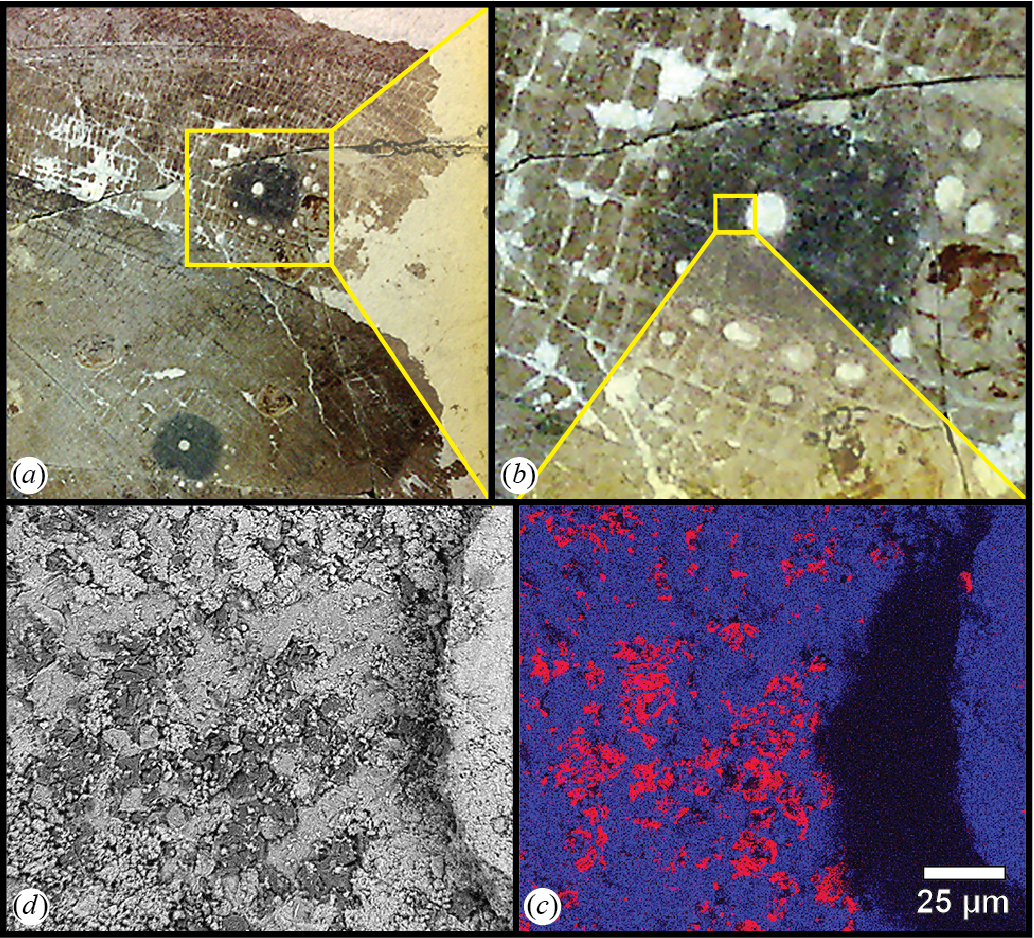


**Figure S2.Light Microscopic and SEM Images with X-ray data for characterizing wing eyespots of a kalligrammatid specimen (*above*).** (***a***) Reflected light micrograph of the wing eyespot region of *Kalligramma circularia* (CNU-NEU-NN-2011-379C), illustrating the pigmentation structure of an ocule and surrounding area of a Type 4 wing eyespot. (***b***) Close-up of an upper eyespot. (***c***) SEM backscattered electron image of a boundary between the central white ocule and pigmented area. (***d***) Composite X-ray map of (*c*) on the left aspect. Absence of an EDS signal adjacent to the ocule is probably due to microtopographical interference. Colors: blue, silicon distribution; red, carbon distribution. Note the absence of carbon within the central white ocule.

**
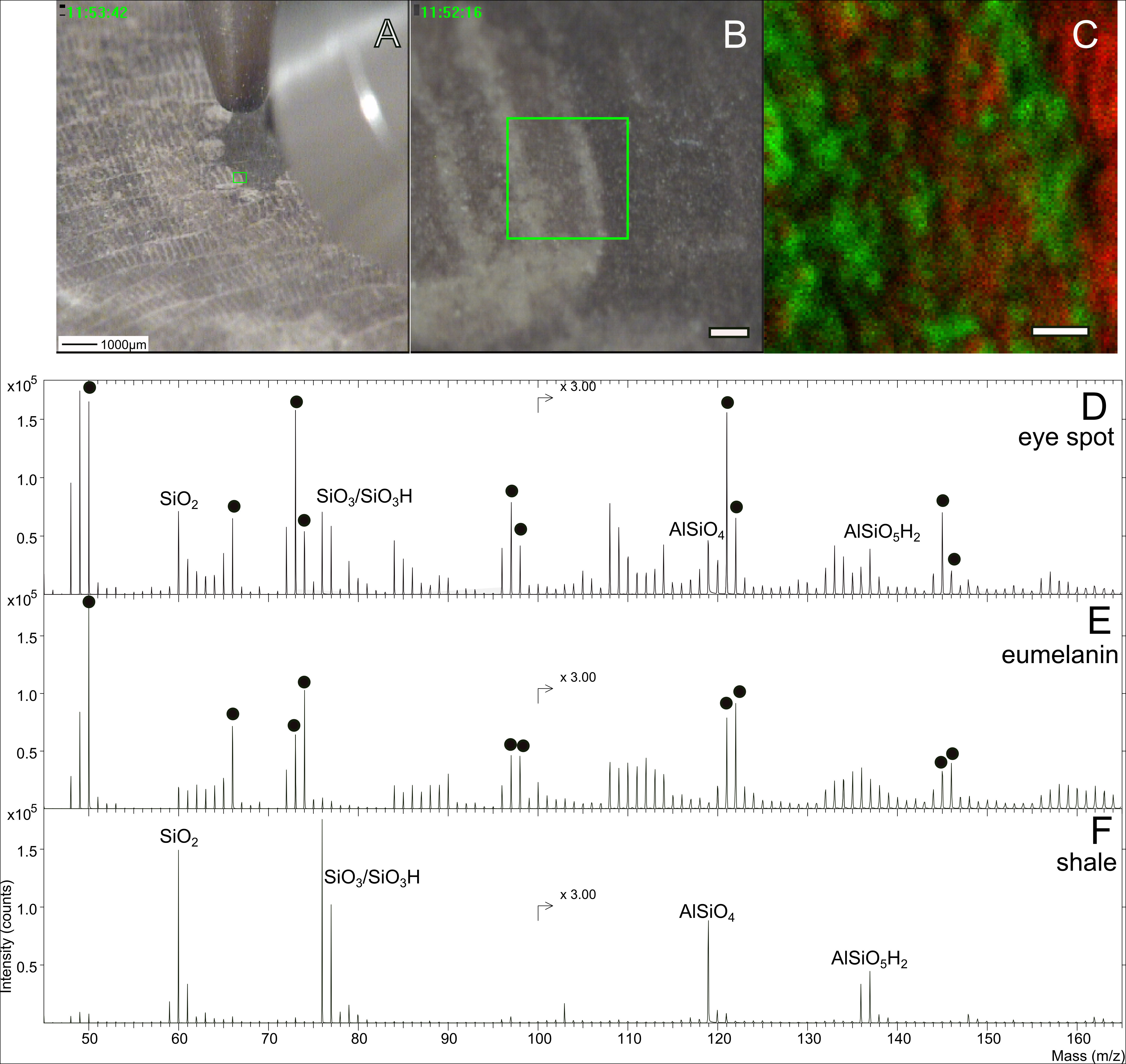
**

**Figure S3. Time-of-flight–secondary ion mass spectrometry (ToF-SIMS) video and ion images, and spectra of the eyespot of *Kallihemerobius feroculus*** **compared to a eumelanin standard and the surrounding matrix** (***above***). (***a***) A ToF-SIMS macro video image of the eyespot. (***b***) A ToF-SIMS micro video image of same eyespot as in (*a*). A green square indicates the area of analysis. Scale bar is 100 µm. (***c***) A ToF-SIMS negative ion image overlay of peaks assigned to eumelanin (green, added *m/z* 50.00, 66.00, 73.01, 74.01, 97.01, 98.00, 121.01, 122.01, 145.00 and 146.00) and to mineral matrix (red, added *m/z* 59.97, 75.96 and 76.97). Scale bar is 50 µmm. ToF-SIMS negative spectra (*m/z* 45–165) of (***d***) eye spot, (***e***) eumelanin (synthetic, Sigma-Aldrich M8631) and (***f***) the shale matrix surrounding the fossil. Peaks assigned to eumelanin are marked with filled circles.

**
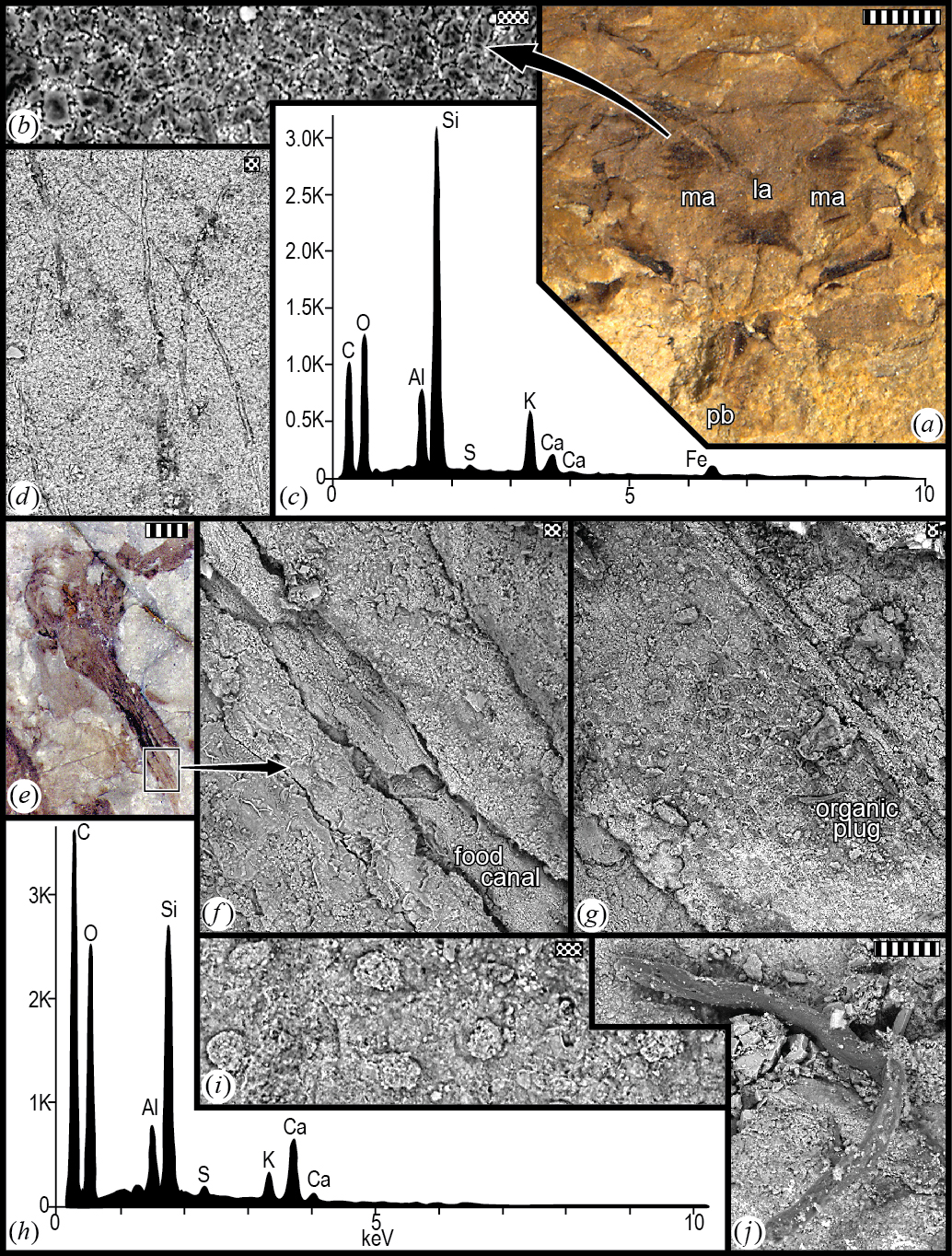
**

**Figure S4 (*above*). Light microscopic and SEM Images with EDS data characterizing mandibulate and siphonate mouthparts of two kalligrammatid specimens (*above*).** The mouthparts of two kalligrammatid specimens are explored: the surface of rudimentary mandibles (*a*–*d*) and the other detailing the contents of material within a proboscis food tube (*e*–*j*). At top, (*a*–*d*) is the specimen and EDS elemental spectra of the right mandible surface from the head underside in *Kallihemerobius feroculus* (CNU-NEU-NN-2010-013P). (***a***), Light microscope image of the head ventral aspect with right mandible (**ma**) surface enlarged at left in (***b***), and elemental EDS spot analysis in (***c***). The labial plate (**la**) and proboscis base (**pb**), with median suture, are indicated as positional reference marks. (***d***), Isolated setae that contrast with the matted hairs found in (*b*). At bottom, (*e*–*j*) are EDS data of the food canal contents from the proboscis of *Kalligramma brachyrhyncha* (CNU-NEU-NN-2009-030P). (***e***), Light photograph of head and proximal proboscis. (***f***), SEM enlargement of food canal within the proboscis. (***g***), SEM backscattered image of a thick organic plug. (***h***), Point chemical analysis of organic plug. (***i***), SEM of two setal socket rows on a nearby cuticular surface. (***j***), SEM of unknown hair-like structures. Scale bars: solid = 10 mm, striped = 1 mm, stippled = 10 µm.

**
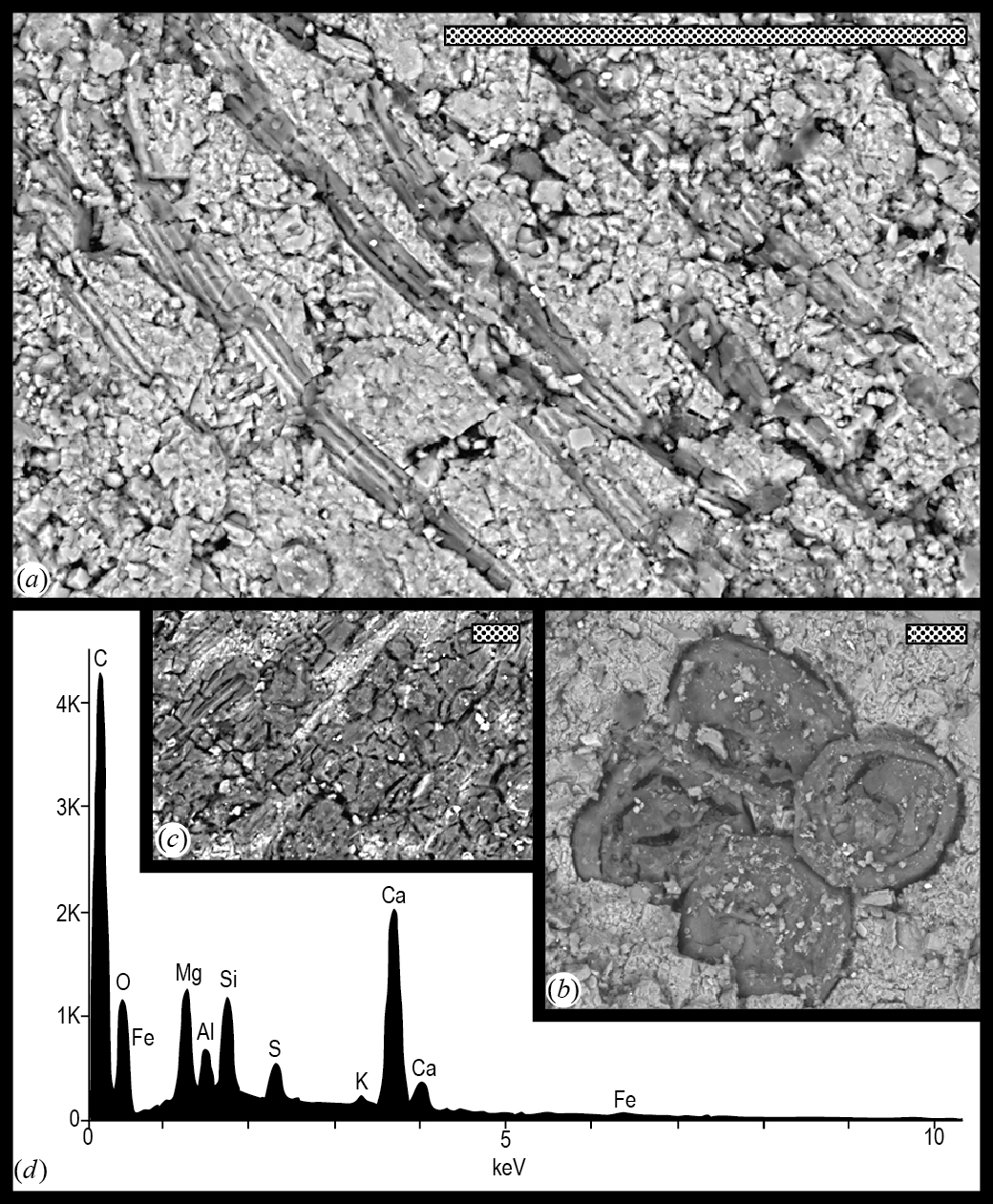
**

**Figure S5 (*above*). Electron microprobe analysis of pollen at the palpal tips of *Meioneurites spectabilis* holotype (PIN-2784/1069), from the Late Jurassic of Karatau of eastern Kazakhstan (*above*).**(***a***), Several five-ridged palpal scales. (***b***), SEM of a *Classopollis* cf. *annulatus* tetrad in matrix adjacent the maxillary palp base. (**c**), Cracked surface of distal maxillary palp. (***d***), Elemental spectrum displaying comparatively high carbon concentrations in (*c*) above. A much less magnified version of the head and mouthparts of this specimen is shown in Fig. S1*.*

**
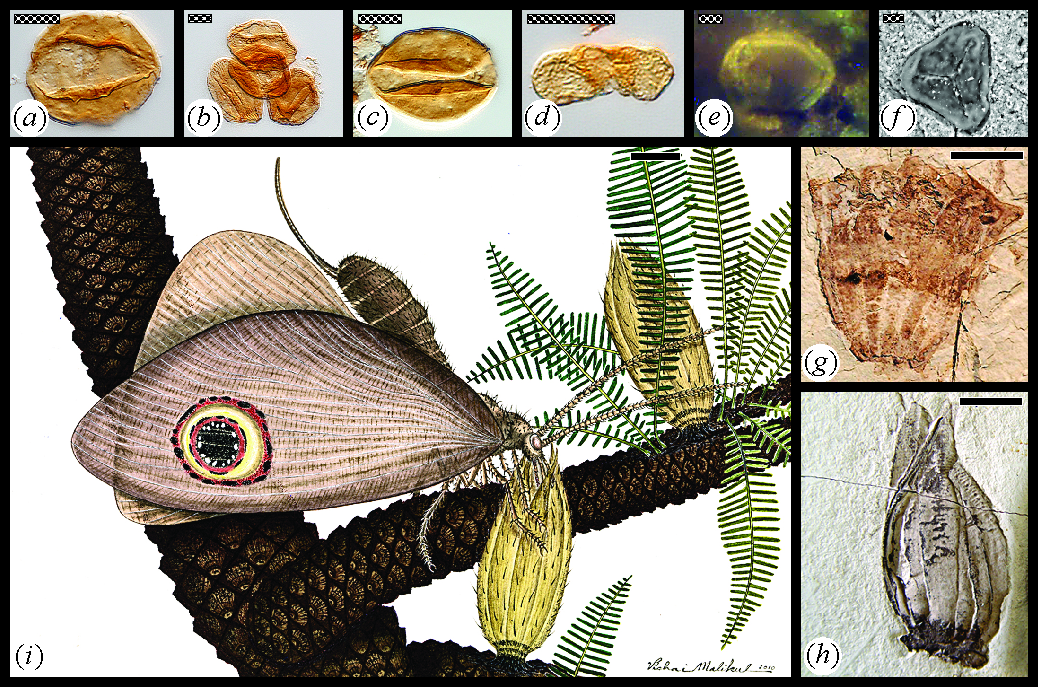
**

**Figure S6 (*above*).** **Plant associations of kalligrammatids.** *a*–*f* are palynomorphs associated with kalligrammatid taxa; (***a***) cf. *Chasmatosporites* of possible Cycadales affiliation; (***b***) tetrad of *Classopollis* cf. *C*. *annulatus* of the extinct conifer Cheirolepidaceae; (***c***) *Cycadopites nitidus* attributable to Bennettitales, Cycadales, Czekanowskiales, Ginkgoales or Pentoxylales; (***d***) *Vitreisporites pallidus* of Caytoniales; (***e***) epifluorescence image of a *Classopollis* cf. *C*. *annulatus* on a foreleg tarsus of *Meioneurites spectabilis* (KAR); and (***f***) gleicheniaceous fern spore. *a*–*e* are pollen macerated from sedimentary matrix adjacent to *Kallihemerobius* *feroculus* (JIU), detailed in figures 4*t* and S2*a*–*d*. (***g***) The bennettitalean strobilus *Weltrichia* sp. (♂, CNU-PLA-LY-2011-001,YIX). (***h***) The earlier occurring bennettitalean strobilus *Williamsonia* sp. (♀, CNU-PLA-NN-2007-018, JIU). (***i***) Reconstruction of specimen from Fig. 1*l*, on a bennettitalean host and probing a *Williamsonia*. Scale bars: solid, 10 mm; striped, 1 mm; dotted, 10 µm.

**Data Supplement References**

1. Handlirsch A.1906/1908 *Die fossilien Insekten und die Phylogenie der rezenter Formen*. Leipzig: Wilhelm Engelmann.

2. Ponomarenko AG. 1984 Neuroptera from the Jurassic in eastern Asia. *Paleontol. Zhur.* **1984**(3), 59–69.

3. Walther J. 1904 Die Fauna der solnhofener Plattenkalke. *Denkschr. Med.–Naturwiss. Ges*. *Jena* **11**, 133–214.

4. Yang Q, Wang YJ, Labandeira CC, Shih C, Ren D. 2014 Mesozoic butterfly-like lacewings from China provides phylogenetic insight into the evolution of the Kalligrammatidae. *BMC Evol. Biol.* **14**, 126.

5. Liu Q, Khramov AV, Zhang HC. 2015 A new species of *Kalligramma* Walther, 1904 (Insecta, Neuroptera, Kalligrammatidae) from the Middle–Upper Jurassic of Daohugou, Inner Mongolia, China, *Alcheringa*.**39**, 438-442.

6. Yang Q, Markarkin VN, Ren D. 2014 Two New Species of Kalligramma Walther (Neuroptera: Kalligrammatidae) from the Middle Jurassic of China. *Ann. Entomol. Soc. Am.*107, 917–925.

7 Panfilov DV. 1968 In *Jurassic insects of Karatau*, (ed BB Rohdendorf), pp. 166–174. Moscow: Nauka Press.

8. Zhang JF, Zhang HC. 2003 *Kalligramma jurarchegonium* sp. nov. (Neuroptera: Kalligrammatidae) from the Middle Jurassic of northeastern China. *Orien. Ins.* **37**, 301–308.

9. Ren D, Guo ZG. 1996 On the new fossil genera and species of Neuroptera (Insecta) from the Late Jurassic of northeast China*. Acta Zootax. Sin.* **21**: 461–479.

10. Jazembowski EA. 2001 In *Turnbridge Wells and Rusthall Commons: A history and natural history* (ed ML Rowlands), pp. 48–58. Turnbridge Wells, UK: Turnbridge Wells Museum and Art Gallery.

11. Panfilov DV. 1980 In *Fossil insects of the Mesozoic* (eds VG Dolin, DV Panfilov, AG Ponomarenko, LN Pritykina), pp. 81–111. Kiev: Naukova Dumka, Kiev.

12. Ren D. 2003 Two new Late Jurassic genera of kalligrammatids from Beipiao, Liaoning (Neuroptera: Kalligrammatidae). *Acta Zootax. Sin.* **28**, 105–109.

13. Makarkin VN, Ren D, Yang Q. 2009 Two new species of Kalligrammatidae (Neuroptera) from the Jurassic of China, with comments on their venational homologies. *Ann. Entomol. Soc. Am.* **102**, 964–969.

14. Zhang JF. 2003 Kalligrammatid lacewings from Upper Jurassic of Daohugou Formation in Inner Mongolia, China. *Acta Geol. Sin*. **77**, 141–146.

15. Ren D, Engel MS. 2008 Aethogrammatidae, a new family of lacewings from the Mesozoic of China (Neuroptera, Myrmeleontiformia). *J. Kansas Entomol. Soc.* **81**, 161–167.

16. Yang Q, Markarkin VN, Ren D. 2011 Two new interesting genera of Kalligrammatidae (Neuroptera) from the Middle Jurassic of Daohugou, China. *Zootaxa* **2873**, 60–68.

17 Liu Q, Zheng DR, Zhang Q, Wang B, Fang Y, Zhang HC. 2013 Two new kalligrammatids (Insecta, Neuroptera) from the Middle Jurassic of Daohugou, Inner Mongolia, China. *Alcheringa* **38**, 65‒69.

18. Martynova OM. 1947 Kalligrammatidae (Neuroptera) from the Jurassic shales of Kara-Tau (Kazakhstan SSR). *Dokl. Akad. Nauk SSSR (NS)* **58**, 2055–2058.

19. Ponomarenko AG. 1992 Neuroptera (Insecta) from the Lower Cretaceous of Transbaikalia. *Paleontol*. *J*. **26**, 55–66.

20. Liu Q, Khramov AV, Zhang HC, Jarzembowski ED. 2015 Two new species of *Kalligrammula* Handlirsch,1919 (Insecta, Neuroptera, Kalligrammatidae) from the Jurassic of China and Kazakhstan. *J. Paleontol*. doi: dx.doi.org/10.1017/jpa.2015.25.

21 Handlirsch A. 1919 Eine neue Kalligrammide (Neuroptera) aus dem Solnhofen Plattenkalke. *Senckenbergiana* **1**, 61–63.

22. Ren D, Oswald JD. 2002 A new genus of Kalligrammatid lacewings from the Middle Jurassic of China (Neuroptera: Kalligrammatidae). *Stutt. Beitr. Naturk. B* **317**, 1–8.

23. Engel MS. 2005 A remarkable kalligrammatid lacewing from the Upper Jurassic of Kazakhstan (Neuroptera: Kalligrammatidae). *Trans. Kansas Acad. Sci.* **108**, 59–62.

24. Yang Q, Zhao YY, Ren D. 2009 An exceptionally well-preserved fossil Kalligrammatid from the Jehol Biota. *Chin. Sci. Bull.* **54**, 1732–1737.

25. Bechly G, Makarkin VN. 2016 A new gigantic lacewing species (Insecta: Neuroptera) from the Lower Cretaceous of Brazil confirms the occurrence of Kalligrammatidae in the Americas. *Cretaceous Res.* **58**:135–140.

26. Wakamatsu K, Ito S. 2002 Advanced chemical methods in melanin determination. *Pigm. Cell Res.* **15**, 174–183.

27. Yoshioka S, Kinoshita S. 2006 Structural or pigmentary? Origin of the distinctive white stripe on the blue wing of a *Morpho* butterfly. *Proc. Roy. Soc. B* **273**, 129–134.

28. Lindgren J, Udval P, Sjövall P, Nilsson DE, Engdahl A, Schultz BP, Thiel V. 2012 Molecular preservation of the pigment melanin in fossil melanosomes. *Nat*. *Commun*. **3**: 824.

29. Lindgren J, Sjövall P, Carney RM, Udval P, Gren JA, Dyke G, Schultz BP, Shawkey MD, Barnes KR, Polcyn MJ. 2014 Skin pigmentation provides evidence of convergent melanism in extinct marine reptiles. *Nature* **586** 484–488.

30. Colleary C, Dolocan A, Gardner J, Singh S, Wuttke M, Rabenstein R, Habersetzer J, Shaal S, Feseha M, Clemens M. 2015 Chemical, experimental, and morphological evidence for diagenetically altered melanin in exceptionally preserved fossils. *Proc. Natl. Acad. Sci. U.S.A.* **112**: 12592–12597.

31. Nepi M*, et al*. 2009 Nectar and pollination drops: How different are they? *Ann. Bot.* **104**, 205–219.

32. Krenn HW, Gereben-Krenn B, Steinwender BM, Popov A. 2008 Flower-visiting Neuroptera: Mouthparts and feeding behaviour of *Nemoptera sinuata* (Nemopteridae). *Euro. J. Entomol.* **105**, 267–277.

33. Tjeder B. 1967 The lace-wings of southern Africa. 4. Family Nemopteridae. *So. Afr. Anim. Life* **13**, 290–501.

34. Labandeira CC, Kvaček J, Mostovski MB. 2007 Pollination drops, pollen, and insect pollination of Mesozoic gymnosperms. *Taxon* **56**, 663–695.

35. Wahlberg H. 2006 That awkward age for butterflies: insights from the age of the butterfly Nymphalinae. *Syst. Biol.* **55**, 703–714.

36. Zhang W, Zheng S. 1987 in *Mesozoic stratigraphy and palaeontology of western Liaoning*, Vol. 3 (eds WL Wang, *et al.*), pp. 239–368. Beijing: Geological Publ. House.

37. Doludenko MP, Orlovskaya ER. 1976 Jurassic floras of the Karatau Range, southern Kazakhstan. *Palaeontology* **19**, 627–640.

38. Sun G, Zheng SL, Dilcher DL, Wang YD, Mei SW. 2001 *Early angiosperms and their associated plants from western Liaoning, China*. Shanghai: Shanghai Scientific and Technological Education Publ. House.

39. Wu Z, Zheng SL. 1987 in *Mesozoic Stratigraphy and Fossils from Western Liaoning, Northeast China*, eds Wang WL; et al.), pp. 239–248. Beijing: Geologic Publ. House.

40 Traverse A. 2007 *Paleopalynology*, Second Edition. Dordrecht, Netherlands: Springer.

41. Friis EM, Pedersen KR. 1996 *Eucommiitheca*, a new pollen organ with *Eucommiidites* pollen from the Early Cretaceous of Portugal. *Grana* **35**, 104–112.
